# A Nonsense Mutation in *TMEM95* Encoding a Nondescript Transmembrane Protein Causes Idiopathic Male Subfertility in Cattle

**DOI:** 10.1371/journal.pgen.1004044

**Published:** 2014-01-02

**Authors:** Hubert Pausch, Sabine Kölle, Christine Wurmser, Hermann Schwarzenbacher, Reiner Emmerling, Sandra Jansen, Matthias Trottmann, Christian Fuerst, Kay-Uwe Götz, Ruedi Fries

**Affiliations:** 1 Lehrstuhl fuer Tierzucht, Technische Universitaet Muenchen, Freising, Germany; 2 Department of Urology, University of Munich, Munich, Germany; 3 ZuchtData EDV-Dienstleistungen GmbH, Wien, Austria; 4 Institut fuer Tierzucht, Bayerische Landesanstalt für Landwirtschaft, Poing, Germany; University of Bern, Switzerland

## Abstract

Genetic variants underlying reduced male reproductive performance have been identified in humans and model organisms, most of them compromising semen quality. Occasionally, male fertility is severely compromised although semen analysis remains without any apparent pathological findings (*i.e.*, idiopathic subfertility). Artificial insemination (AI) in most cattle populations requires close examination of all ejaculates before insemination. Although anomalous ejaculates are rejected, insemination success varies considerably among AI bulls. In an attempt to identify genetic causes of such variation, we undertook a genome-wide association study (GWAS). Imputed genotypes of 652,856 SNPs were available for 7962 AI bulls of the Fleckvieh (FV) population. Male reproductive ability (MRA) was assessed based on 15.3 million artificial inseminations. The GWAS uncovered a strong association signal on bovine chromosome 19 (P = 4.08×10^−59^). Subsequent autozygosity mapping revealed a common 1386 kb segment of extended homozygosity in 40 bulls with exceptionally poor reproductive performance. Only 1.7% of 35,671 inseminations with semen samples of those bulls were successful. None of the bulls with normal reproductive performance was homozygous, indicating recessive inheritance. Exploiting whole-genome re-sequencing data of 43 animals revealed a candidate causal nonsense mutation (*rs378652941*, c.483C>A, p.Cys161X) in the transmembrane protein 95 encoding gene *TMEM95* which was subsequently validated in 1990 AI bulls. Immunohistochemical investigations evidenced that TMEM95 is located at the surface of spermatozoa of fertile animals whereas it is absent in spermatozoa of subfertile animals. These findings imply that integrity of TMEM95 is required for an undisturbed fertilisation. Our results demonstrate that deficiency of TMEM95 severely compromises male reproductive performance in cattle and reveal for the first time a phenotypic effect associated with genomic variation in *TMEM95*.

## Author Summary

Impaired male fertility is a prevalent condition in many species and is often explained by aberrant semen quality. In some cases, male fertility is severely compromised although semen quality is without any apparent pathological findings (*i.e.*, idiopathic male subfertility). The genetic mechanisms underlying idiopathic male subfertility often remain unexplained. In the present paper, we report a recessively inherited variant of idiopathic male subfertility in a cattle population. We use 650,000 genome-wide SNP markers genotyped in >7900 artificial insemination bulls to pinpoint the underlying genomic region. We take advantage of whole-genome re-sequencing data of 43 animals to identify a causal loss-of-function mutation in *TMEM95* encoding a nondescript transmembrane protein. We demonstrate that transmembrane protein 95 is located at the plasma membrane of spermatozoa of fertile animals whereas it is absent in spermatozoa of subfertile animals. Our results indicate that integrity of transmembrane protein 95 is required for an undisturbed fertilisation. This is the first report to reveal a phenotypic effect associated with genomic variation in *TMEM95* in any organism.

## Introduction

Impaired reproductive performance is a prevalent condition in both sexes of many species and up to 15% of couples are affected in humans [1], [2]. The disability to reproduce is defined as infertility (*i.e.*, sterility), whereas subfertility refers to any form of reduced fertility [3].

Low sperm concentration (*i.e.*, oligospermia) and the absence of spermatozoa (*i.e.*, azoospermia), respectively, are frequently diagnosed in males with impaired fertility [4]. Further aberrant semen quality traits (*e.g.*, abnormal sperm morphology [5], reduced motility [6], [7]) account for another substantial fraction of reduced male fertility. However, semen analysis of a considerable number of males with impaired reproductive performance remains without any apparent pathological findings (*i.e.*, unexplained/idiopathic infertility) [8], [9].

Semen quality traits have low to medium heritability in cattle populations [10]. Numerous genetic variants underlying routinely assessed semen quality traits have been identified so far in humans [11], [12], model species [13] and livestock populations [14]. However, the number of known genetic mechanisms causing idiopathic male subfertility is very small [15], [16] and identified polymorphisms explain only a small fraction of its genetic variation [17].

Artificial insemination (AI) is predominant over natural service in most cattle populations and all ejaculates are closely examined immediately after semen collection. Only semen samples without any apparent abnormalities, such as low sperm count, reduced progressive motility, low viability, abnormal morphology of spermatozoa, are used for insemination. However, the reproductive performance indicated by the proportion of successful inseminations varies considerably among AI sires [18], [19]. So far, genome-wide association studies (GWAS) for male reproductive traits were of limited success in cattle populations [20], [21] and only one putatively causative mutation has been identified [22].

Here we report a new recessively inherited variant of idiopathic male subfertility in the Fleckvieh (FV) cattle population. The mapping of the underlying genomic region was facilitated by using high-density genotypes in a large sample of artificial insemination bulls with phenotypes for reproductive performance assessed based on 15 million artificial inseminations. Exploiting whole-genome re-sequencing data revealed a causative loss-of-function mutation in the transmembrane protein 95 encoding gene *TMEM95*.

## Results

### Male subfertility in the Fleckvieh cattle population

Phenotypes for male reproductive ability (MRA) were obtained for 7962 bulls of the FV population based on 15.3 Mio artificial inseminations (AI). The values for MRA range from −40 to +13 and reflect the bulls' reproductive performance as percentage deviation from the population mean. Male reproductive ability is highly correlated (r = 0.59) with the 56-day non-return rate (NRR56) in cows. The NRR56 is the proportion of cows that are not re-inseminated within a 56-day interval after the first insemination. After visual inspection of the distribution of MRA, forty-nine bulls with exceptionally poor reproductive performance (MRA<−20) were considered as subfertile (Figure S1 and Table 1). Animals with values for MRA below −20 ( = five standard deviations below the population mean) were used as case group in a case-control design.

**Table 1:** Characteristics of the case/control design.

Group	Phenotype	N	MRA	NRR56
Control	Normal reproductive performance	7913	−0.44 (±3.16)	65.46 (±5.35)
Case	Subfertile	49	−27.69 (±4.15)	29.16 (±5.27)

The 7962 artificial insemination (AI) bulls were subdivided based on their male reproductive ability (MRA). Forty-nine bulls with MRA≤−20 were considered as subfertile. The mean and standard deviation for MRA and the 56-day non-return rate (NRR56) is presented for both groups.

### Bovine male subfertility maps to chromosome 19

**Figure 1:**
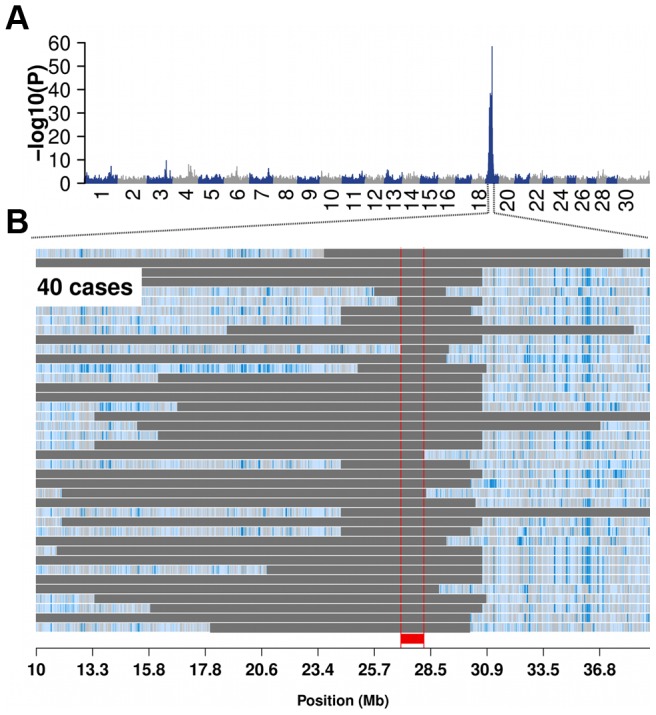
Bovine male subfertility maps to chromosome 19 in the Fleckvieh cattle population. Association of 652,856 SNPs with male reproductive ability (MRA) in 7962 FV bulls (A). P-values were obtained by fitting a linear mixed model. Autozygosity mapping in 40 subfertile bulls (B). Blue and pale blue represent homozygous genotypes (AA and BB), heterozygous genotypes (AB) are displayed in light grey. The solid grey bars represent segments of extended homozygosity in 40 subfertile bulls. The red bar indicates the common segment of homozygosity. The shared segment of homozygosity encompasses 80 transcripts, among them *TMEM95*. The full list of genes within the 1386 kb segment is presented in Table S1.

Using MRA as quantitative trait in a genome-wide association study (GWAS) yielded a strong association signal on bovine chromosome (BTA) 19 (P = 4.38×10^−20^, Figure S2). However, the association signal was more pronounced using 49 subfertile animals (MRA<−20) as case group and the remaining 7913 animals as controls (Table 1 and Figure 1A). The most significantly associated SNP is located at 30,220,186 bp (*ARS-BFGL-NGS-11488*; P = 4.08×10^−59^).

Autozygosity mapping revealed a common 1386 kb segment (26,580,096 bp–27,956,634 bp) of extended homozygosity in 40 subfertile bulls containing 80 genes (Figure 1B and Table S1). None of 7913 bulls with normal reproductive performance was homozygous for the 1386 kb segment, indicating recessive inheritance. Semen samples of 40 homozygous bulls had been used for 35,671 artificial inseminations with an average of 892 inseminations per bull. This is a typical number for test inseminations performed with semen samples of young bulls in progeny testing based breeding programmes. However, only 619 (1.74%) of those inseminations were successful (Table S2).

There was no evidence for the presence of large structural variants (i.e., copy number variations) within the segment of extended homozygosity (Figure S3). The proportion of missing genotypes did not significantly differ between cases and controls (P>0.09) for all SNPs located within the associated region.

**Figure 2:**
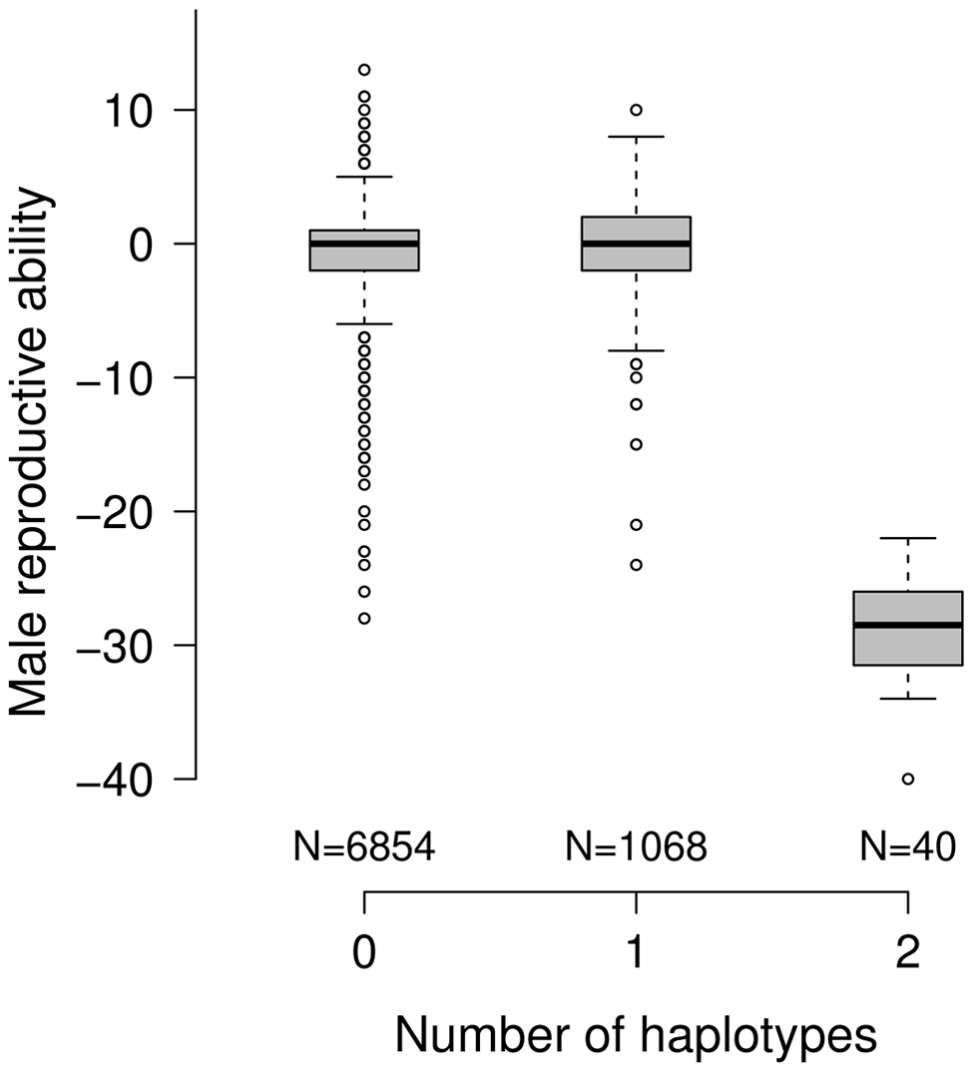
Effect of the subfertility-associated haplotype on male reproductive ability. The boxplots display the male reproductive ability (MRA) of 7962 artificial insemination bulls as a function of copies of the subfertility-associated haplotype. The reproductive performance of heterozygous bulls (N = 1068) is normal, whereas MRA is <−20 for homozygous bulls (N = 40).

The frequency of the subfertility-associated haplotype amounts to 7.2%. Of 7962 genotyped bulls with phenotypes for MRA, 1068 (13.41%) carry the deleterious haplotype in heterozygous state. The carrier frequency increased considerably within the last years (P = 0.0002, Figure S4). The reproductive performance of heterozygous bulls is normal, indicating recessive inheritance (Figure 2). Of 1952 primiparous cows, 291 are heterozygous and 17 are homozygous for the subfertility-associated haplotype. The haplotype neither affects reproductive performance nor milk production traits in females (Table S3). The haplotype distribution does not deviate from the Hardy-Weinberg equilibrium, neither in females (P = 0.303) nor in males (P = 0.817).

Both, haplotype and pedigree analysis allowed to trace the mutation back to the bull *HAXL* (*1966) (Figure S5). *HAXL* appears in the pedigrees of 7779 out of 7962 bulls (97.70%) and can be considered as the most important ancestor of the current FV population [23].

### Exploiting whole-genome re-sequencing data for mutation detection

**Figure 3:**
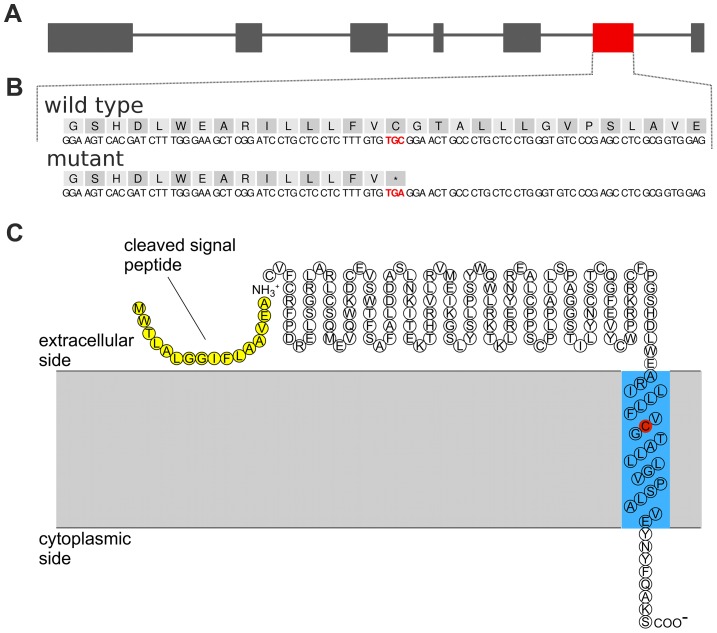
A nonsense mutation in *TMEM95* resides within the predicted transmembrane domain of the encoded transmembrane protein 95. Genomic structure of the transmembrane protein 95 encoding gene *TMEM95* (A). Grey and red boxes represent exons. The red box represents exon 6 including *rs378652941*, introducing a premature stop codon. Genomic and protein sequence of exon 6 of *TMEM95* (B). The affected codon (p.Cys161X, TGC→TGA) is highlighted with red colour. Predicted protein topology of TMEM95 (C). TMEM95 is a single-pass type I transmembrane protein with a predicted N-terminal signal peptide sequence (yellow) and a 23-amino acid transmembrane domain (blue). The affected codon (p.Cys161X) resides within the predicted transmembrane domain (red).

Whole genome re-sequencing of 43 animals and subsequent multi-sample variant calling yielded genotypes at 17.17 million sites [23]. Among them, 5965 (5287 SNPs and 678 INDELs) are located within the subfertility-associated region on BTA19 (26,580,096 bp to 27,956,634 bp). Six of the 43 re-sequenced animals were identified as carriers of the associated haplotype *via* high-density genotypes. The sequence data were filtered for variants compatible with the supposed recessive inheritance, *i.e.*, heterozygous in carriers and homozygous for the reference allele in non-carriers (see Material & Methods, Figure S6). After filtering, 26 SNPs and six INDELs were retained as candidate causal mutations (Table S4 and S5). The functional effects of those variants were predicted based on the gene annotation of the UMD3.1 assembly of the bovine genome [24]. Four of the 32 compatible variants were located in coding regions (Table 2). Among them, we considered a nonsense mutation in *TMEM95* (*rs378652941*, c.483C>A, p.Cys161X, Chr19: 27,689,622 bp) as the prime candidate causal mutation (Figure 3A and 3B). The nonsense mutation was subsequently confirmed in the re-sequenced animals by classical Sanger sequencing (Figure S7 and S8).

**Table 2:** Coding variants compatible with recessive inheritance.

Chr	Chromosomal position (bp)	NCBI Assay ID	Reference allele	Alternative allele	Affected gene	Effect	*Polyphen-2* prediction
19	27,042,848	rs381722524	T	C	*KIF1C*	p.Gln66Arg	Benign
19	27,299,006	rs208952900	C	T	*PELP1*	p.Phe250Phe	-
19	27,570,146	rs385135118	C	A	*ACADVL*	p.Pro236Thr	possibly damaging
19	27,689,622	rs378652941	C	A	*TMEM95*	p.Cys161X	-

Four compatible polymorphic sites are located in coding regions. The functional annotation of the polymorphisms was obtained based on the UMD3.1 gene prediction [24]. The effect of non-synonymous substitutions was predicted using *Polyphen-2* [75].

### A loss-of-function mutation in *TMEM95* causes male subfertility in cattle

Genotypes for two non-synonymous substitutions in *ACDVL* and *KIF1C* and for the nonsense mutation in *TMEM95* were obtained for cases and controls using TaqMan genotyping assays (Table 3). Only c.483C>A, introducing the premature stop-codon in *TMEM95* (p.Cys161X), was perfectly associated. All animals, which are homozygous for the subfertility-associated haplotype, are homozygous for the non-reference allele, whereas none of 1396 FV bulls with normal reproductive performance are homozygous. The polymorphism is present in the FV breed only; 277 Holstein-Friesian and 278 Braunvieh animals are homozygous for the reference allele. The c.483C>A-mutation is not segregating among 15 Jersey, 47 Angus and 129 Holstein-Friesian animals which were sequenced in the context of the 1000 bull genomes project [25].

**Table 3:** Validation of three coding variants.

	*rs381722524*			*rs385135118*			*rs378652941*		
	*KIF1C* – p.Gln66Arg			*ACADVL* – p.Pro236Thr			*TMEM95* – p.Cys161X		
	CC	CT	TT	AA	AC	CC	AA	AC	CC
FV_subfert_	39	-	-	39	-	-	39	-	-
FV_ctrl_	1	74	650	6	103	606	-	176	1220
BV	-	1	189	-	3	181	-	-	278
HF	-	23	245	-	-	268	-	-	277

Genotypes for three coding variants were obtained using TaqMan genotyping assays. The genotypes are presented for FV animals identified as homozygous for the subfertility-associated haplotype (FV_subfert_) and for FV animals with normal reproductive performance (FV_ctrl_). Note that DNA was available for 39 out of 40 subfertile FV bulls only. Additionally, genotypes were obtained for randomly selected Braunvieh (BV) and Holstein-Friesian (HF) animals with normal reproductive performance.

*TMEM95* encodes a highly conserved single-pass type I transmembrane protein consisting of 183 amino acids with a predicted extracellular N-terminal signal peptide, a 23-amino acid transmembrane domain (amino acid position 153 to 175) and a 8-amino acid intracellular C-terminal domain (Figure 3C and Figure S9 and S10). The premature stop codon introduced by the c.483C>A-mutation is located within the predicted transmembrane domain and truncates the protein by 22 amino acids.

### The c.483C>A-mutation does not affect semen quality

Semen quality (morphology, vitality, total motility) was analysed using cryopreserved semen samples of 30 bulls (10 *wt/wt*, 10 *wt/mt*, 10 *mt/mt*). In all ejaculates, spermatozoa showed less than 20% morphological alterations and less than 5% morphological alterations of the head. Total motility after thawing ranged from 50 to 65%. Statistical analysis showed no significant differences in the proportion of motile spermatozoa from *wt/wt*, *wt/mt* and *mt/mt* bulls (Table 4). As shown by eosin staining, 40 to 70% of the spermatozoa were viable after thawing. There were no significant differences in the percentages of viable spermatozoa between *wt/wt*, *wt/mt* and *mt/mt* bulls. Additionally, ejaculate volume, sperm concentration and progressive motility were assessed in fresh semen samples of 203 AI bulls (177 *wt/wt*, 21*wt/mt*, 5 *mt/mt*). Ejaculate volume was above 5 ml, sperm count was above 1.42 Mio/µl and the proportion of spermatozoa with progressive motility was above 70% for all animals (Table 5).

**Table 4:** Assessment of cryopreserved semen quality after thawing.

*rs378652941*	N	Motile spermatozoa (%)	Vital spermatozoa (%)
*wt/wt*	10	53±9	56±11
*wt/mt*	10	61±13	65±11
*mt/mt*	10	48±13	51±12

The mean and standard deviation of the proportion of motile and vital spermatozoa was assessed from cryopreserved (−196°C) sperm specimens of 30 artificial insemination bulls immediately after thawing. Cryopreserved semen samples were obtained from artificial insemination companies.

**Table 5:** Assessment of fresh semen quality.

*rs378652941*	N	Ø number of ejaculates	Ejaculate volume (ml)	Sperm count (Mio/µl)	Spermatozoa with progressive motility (%)
*wt/wt*	177	52	5.14±1.08	1.53±0.22	74.73±5.21
*wt/mt*	21	52	5.15±1.03	1.47±0.23	75.15±4.92
*mt/mt*	5	63	5.56±1.75	1.42±0.29	70.82±7.06

Fresh semen quality of 203 FV bulls. The mean and standard deviation is presented based on an average of 53 ejaculates per bull. The age of the bulls at semen collection ranged from 1.11 to 2.49 years. Semen quality parameters were kindly provided by Bayern Genetik GmbH (http://www.fleckvieh.de).

### Localization of TMEM95 in spermatozoa

**Figure 4:**
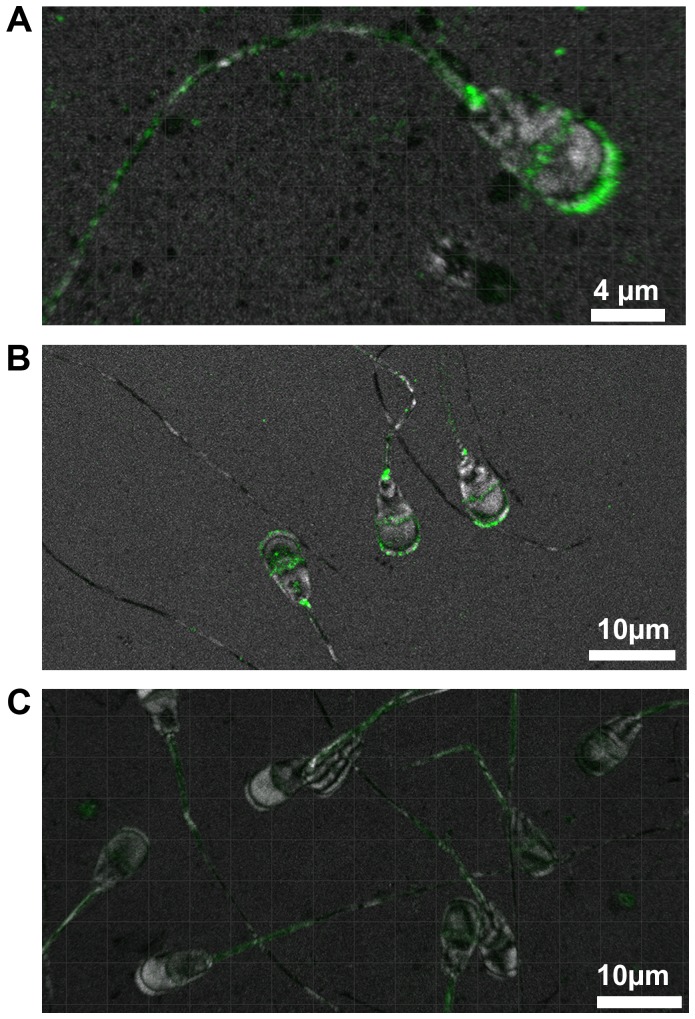
Immunohistochemical localisation of TMEM95. In spermatozoa of *wt/wt* animals, TMEM95 is located at the plasma membrane of the acrosome, on the equatorial segment and at the neck (A). Spermatozoa of *wt/mt* animals show the same fluorescence pattern as spermatozoa of *wt/wt* animals (B). Transmembrane protein 95 is absent in spermatozoa of *mt/mt* animals (C). Note that all spermatozoa exhibit weak fluorescence at the midpiece of the tail due to the autofluorescence of the mitochondria.

A mouse-derived polyclonal antibody generated against human transmembrane protein 95 was used to locate its position in spermatozoa of 33 bulls (10 *wt/wt*, 10 *wt/mt* and 13 *mt/mt*). In spermatozoa of *wt/wt* bulls, TMEM95 was distinctly located on the plasma membrane of the acrosome (Figure 4A). Staining was also visible on the equatorial segment of the head. The sperm neck was regularly labelled. Spermatozoa of *wt/mt* and *wt/wt* bulls showed an identical staining pattern (Figure 4B), whereas spermatozoa of *mt/mt* bulls did not show any staining on the head (Figure 4C). Weak fluorescence was detected in the midpiece of the tail in spermatozoa of all animals due to the autofluorescence of the mitochondria. In the negative controls, there was no signal detectable on the sperm head whereas the midpiece of the tail showed weak autofluorescence (Figure S11).

## Discussion

The genome-wide association study (GWAS) with imputed genotypes for 7962 artificial insemination bulls identified a genomic region on BTA19 for male reproductive ability (MRA) in the FV population. Autozygosity mapping revealed a common 1386 kb segment of extended homozygosity in 40 bulls with unexplained exceptionally poor reproductive performance. None of the bulls with normal reproductive performance was homozygous indicating recessive inheritance. Only 1.74% of inseminations performed with semen samples of affected bulls were successful, although semen quality parameters were within a normal range, reflecting idiopathic subfertility [26]. The newly identified congenital defect is denominated as “*Bovine Male Subfertility*” and accounts for 82% of FV bulls with exceptionally poor reproductive performance. However, we cannot exclude the possibility that homozygous males are infertile and that the very low proportion of successful inseminations reflects errors in parentage recording which might be as high as 10% in dairy cattle breeding programmes [27]. In progeny testing based breeding programmes, semen doses of young bulls are used for approximately 1000 test inseminations [28]. These artificial inseminations are performed within very short time, precluding the early identification of subfertile/infertile bulls. Identifying and culling bulls with poor fertility prognosis (*i.e.*, homozygous bulls) before they are used for artificial insemination is now possible. There was no evidence for any additional genomic region underlying idiopathic male subfertility in the FV population, although the reproductive ability of nine bulls which are not homozygous for the c.483C>A-mutation, is very low. However, the number (n = 9) of subfertile bulls not attributable to the BTA19 locus might not be sufficient for detecting additional loci (Figure S12 and Table S6).

The potential of targeted or whole genome re-sequencing for the identification of causal trait variants has been demonstrated in several species (*e.g.*, [29]–[31]) including cattle [32]–[34]. Causal trait variants for monogenic disorders are traditionally identified by sequencing case/control-panels and by subsequently comparing allele counts in affected and unaffected individuals. However, the concept of the present study is different: the identification of the underlying mutation was based on whole genome re-sequencing data of 43 unaffected FV animals explaining a vast majority of the population's genomic variation [23]. As the frequency of the mutation was reasonably high (7.2%), the affected haplotype was present in heterozygous state in six of the re-sequenced animals. Filtering the re-sequencing data for variants compatible with the supposed recessive inheritance pattern revealed a plausible candidate causative loss-of-function mutation in *TMEM95* encoding the transmembrane protein 95.

The nonsense mutation was perfectly associated in 1990 animals representing three different breeds. To our knowledge, this is the first report of a phenotypic effect associated with variation in *TMEM95* in any organism. So far, there are no clues about the precise function of TMEM95. However, it seems likely that TMEM95 is involved in sperm-egg interactions, which has been shown to be the main function of sperm-specific transmembrane proteins (*e.g.*, [35], [36]). The phenotype in the present study resembles phenotypic patterns of *Caenorhabditis elegans* resulting from an impaired function of sperm-specific transmembrane proteins [37], [38]. Taken together, our findings evidence genomic variation within *TMEM95* to severely compromise the reproductive performance in cattle.

The causative polymorphism (c.483C>A, *rs378652941*) introduces a premature stop codon in *TMEM95* (p.Cys161X). The affected codon resides within the predicted transmembrane domain of TMEM95 most likely resulting in a disturbed anchorage of the truncated protein in the sperm plasma membrane bilayer. It is also likely that the resulting truncated protein is absent due to nonsense-mediated mRNA decay [39]. Our data show no evidence that the mutation affects any of the routinely assessed semen quality parameters in vitro. However, we cannot exclude the possibility that the mutation affects semen quality parameters, *e.g.*, vitality and motility, *in vivo* [40], [41].

Transmembrane protein 95 is primarily located on the acrosomal membrane of the sperm head indicating that it may be involved in the acrosome reaction. Spermatozoa of *mt*/*mt* animals showed no fluorescence at the acrosomal membrane implying deficiency of TMEM95. Thus, successful fertilization by spermatozoa of *mt*/*mt* animals might be compromised. This is supported by the fact that the equatorial segment of the acrosome, which provides the first contact of the spermatozoon with the cell membrane of the oocyte [42], is also labelled in spermatozoa of fertile animals. The sperm neck contains the centriole and is essential for cell division and development of the early embryo [43]. Labelling of the neck indicates an additional potential role of TMEM95 after fertilization during the first cell divisions of the early embryo.

Although spermatozoa of *mt*/*mt* animals showed fluorescence, neither on the acrosomal membrane and the equatorial segment nor at the centriole, weak unspecific fluorescence was observed at the midpiece of the tail. This fluorescence pattern is also present in spermatozoa of w*t*/w*t* and w*t*/*mt* animals. The midpiece is the only region of spermatozoa that contains mitochondria [44]. The weak fluorescence of the midpiece is due to unspecific autofluorescence of the mitochondria, which has been described in several organs and species [45]–[47].

Male subfertility is also present in other species besides cattle [9], [48]–[50], and compromised sperm surface proteins account for a substantial number of males with distinctly reduced reproductive ability in humans [15], [40]. Our results demonstrate that TMEM95 is another sperm surface protein, which is likely to be involved in sperm-egg plasma membrane interactions. Its protein sequence is highly conserved among species (Figure S10) and genetic variants disrupting TMEM95 are likely to induce male subfertility also in other species than cattle. Numerous polymorphic sites have been identified in human *TMEM95*, among them several potential loss-of-function variants (Figure S13). Based on our findings it is highly recommended to systematically survey variants in *TMEM95* as potentially causal for idiopathic male in- or subfertility in any species.

Frequencies of variants that disrupt protein-coding genes are usually low in human populations [51], [52]. However, in livestock populations, the frequency of deleterious alleles might increase rapidly because individual sires can generate tens of thousands of progeny by artificial insemination (*e.g.*, [53], [54], [32]). The loss-of-function mutation in *TMEM95* can be traced back to *HAXL* (*1966), the most important ancestor of the current FV population. Within eight generations, the frequency of the deleterious allele increased to 8.9% and 1443 (13.92%) animals of the present study are heterozygous. This increase of the allele frequency had been possible because phenotypic effects become apparent in homozygous males only. There are no phenotypic effects detectable neither in heterozygous nor in homozygous females (Table S3).

In agreement with previous findings in livestock [55] and humans [15], our results evidence that standard assessment of spermatozoa (*i.e.*, morphology, motility and vitality) is not sufficient to reliably anticipate male reproductive performance. All routinely assessed semen parameters of bulls homozygous for the nonsense mutation in *TMEM95* comply with current requirements for artificial insemination in cattle [56]. It might be advisable to develop functional assays, *e.g.*, for the integrity of sperm surface proteins, for an efficient prospective monitoring of male fertility.

## Materials and Methods

### Ethics statement

Semen samples were collected by approved commercial artificial insemination stations as part of their regular breeding and reproduction measures in cattle industry. No ethical approval was required for this study.

### Animals and phenotypes

Male reproductive ability (MRA) was evaluated in 7962 AI bulls of the German FV population. Semen samples of those bulls were used for 15,321,171 artificial inseminations with an average of 1924 artificial inseminations per bull. The phenotypes for MRA were obtained from routine breeding value estimation for reproductive traits, which are jointly estimated for males and females [57]. The resulting phenotypes for MRA represent the bulls' reproductive performance adjusted for environmental and genetic effects (*i.e.*, year, season, flock, female mating partner). The lower the value for MRA, the worse is the bull's reproductive performance (*i.e.*, the smaller the proportion of successful inseminations).

### Genotypes, quality control and genotype imputation

A total of 3545 animals (1475 AI bulls, 2070 primiparous cows) of the FV population were genotyped with the Illumina BovineHD Bead chip comprising 777,962 SNPs. Another 7073 AI bulls were genotyped with the BovineSNP50 Bead chip comprising ∼54,000 SNPs. The chromosomal position of the SNPs was determined according to the UMD3.1 assembly of the bovine genome [58]. Mitochondrial, Y-chromosomal and those SNPs with unknown chromosomal position were not considered for further analyses. Stringent quality control was carried out for each dataset separately using *PLINK* v1.07 [59]. Animals and SNPs with call-rate <0.95 were excluded, as well as SNPs with minor allele frequency <0.5% and those SNPs deviating significantly from the Hardy-Weinberg equilibrium (P<10^−6^). The pairwise genomic relationship [60] was compared with the pedigree relationship tracing pedigree records back to 1920 [61]. Animals showing major discrepancies of the pedigree and the genomic relationship were removed from the dataset, as such patterns indicate sample swaps. After quality control, the high-density dataset contained 3332 animals and 652,856 SNPs with an average per-individual call-rate of 99.17%. The medium-density dataset contained 7031 animals and 42,907 SNPs with an average per-individual call-rate of 99.75%. Genotype imputation was performed to extrapolate medium-density genotypes to higher density using a pre-phasing based approach. Haplotypes were inferred for the two datasets separately using *Beagle* [62] and subsequent haplotype-based imputation was performed with *Minimac* [63]. This approach yields high imputation accuracy in cattle [64]. The imputed dataset comprised 10,363 animals (8411 AI bulls/1952 primiparous cows) and genotypes for 652,856 SNPs. Phenotypic records for MRA were available for 7962 bulls.

### Genome-wide association study

Genome-wide association studies were performed applying a variance component based approach to account for population stratification. We used *EMMAX* [65] to fit the mixed model 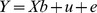, where Y is a vector of phenotypes, b is the SNP effect, X is a design matrix of imputed SNP genotypes, u is a vector of additive genetic effects assumed to be normally distributed with mean 0 and (co)variance 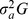, with 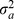 being the additive genetic variance and G being the realized genomic relationship matrix (GRM) of the 7962 bulls with phenotype information built based on 635,224 autosomal SNPs (see above) and where e is a vector of random normal deviates 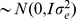.

### Exploiting whole genome re-sequencing data for mutation screening

The genomes of 42 key and contemporary animals of the FV population were sequenced at low- to medium coverage (ø 7.4-fold) and one animal was sequenced at high coverage (25-fold) using Illumina GA IIx and HiSeq 2000 instruments [66], [23]. Paired-end reads were obtained and mapped to the bovine reference sequence (UMD3.1 [58]) using the Burrows-Wheeler Aligner (*BWA*) [67]. *PICARD* (http://picard.sourceforge.net) was used to mark PCR-duplicates. Subsequent multi-sample variant calling with *mpileup* [68] yielded genotypes at 17.17 million sites. The re-sequencing data were contributed to the 1000 bull genomes project [25] and all variants were submitted to dbSNP [23]. *Beagle* phasing and imputation within the 43 sequenced animals improved the primary genotype calls (a detailed overview of the entire variant calling pipeline and all obtained variants is presented in Jansen et al. [23]). Of 17.17 million sites, 5287 SNPs and 678 INDELs were located within the 1386 kb segment (26,580,096 bp to 27,956,634 bp) of extended homozygosity on BTA19. Of the 43 sequenced animals, six were identified as carriers of the subfertility-associated haplotype *via* high-density genotypes, among them the animal sequenced at high coverage. Assuming perfect correlation between the subfertility-associated haplotype and the causal mutation, the allele frequency of the causal mutation should be 7% (6 of 86 affected alleles) in the sequence-derived genotypes. To account for inaccurately genotyped variants due to the low-coverage sequence data (*e.g.*, mis-calling of heterozygous genotypes for rare variants [69]) [70] and for possible phasing errors, a conservative mutation scan was performed to identify variants compatible with recessive inheritance (Figure S6). The 5965 polymorphic sites were filtered for variants that met three conditions: (i) the frequency of the non-reference allele is below 10%, (ii) the variant is heterozygous in the animal sequenced at high coverage and (iii) the variant is present in heterozygous state in at least three of five carrier sequenced at low coverage.

### Validation of three identified polymorphisms

PCR primers (5′-CACCCTGCCTTGTCTTTCAT-3′ and 5′-AGGCTCTGTCCTCGTTTTCA-3′) were designed for exon 6 of *TMEM95* to scrutinize the *rs378652941*-polymorphism by classical Sanger sequencing in the re-sequenced animals as recommended by Jansen et al. [23]. Genomic PCR products were sequenced using the BigDye Terminator v1.1 Cycle Sequencing Kit (Life Technologies) on the ABI 3130x1 Genetic Analyzer (Life Technologies). Genotypes for *rs378652941* (*TMEM95*:c.483C>A, p.Cys161X, Chr19:27689622), *rs381722524* (*KIF1C*:c.197A>G, p.Gln66Arg, Chr19:27042848) and *rs385135118* (*ACADVL*:c.706C>A, p.Pro236Thr, Chr19:27570146) were obtained by TaqMan genotyping assays (Life Technologies) in 1990, 1222 and 1206 animals, respectively, representing three different breeds (BV, FV, HF). The primer and probe sequences are listed in Table S7.

### Topology prediction for TMEM95

The topology of bovine transmembrane protein 95 (NCBI reference sequence: XP_002695846.1) was predicted with *SPOCTOPUS* [71] and *PHOBIUS* [72]. Both methods predict the transmembrane protein topology while accounting for the existence of a N-terminal signal peptide sequence. The protein topology was visualized with *SOSUI* [73]. *ClustalW* [74] was used for multi-species alignment of the protein sequence and for the prediction of conserved regions.

### Assessment of sperm morphology, motility and viability

Cryopreserved (−196°C) sperm specimens of 30 bulls (10 *wt/wt*, 10 *wt/mt*, 10 *mt/mt*) were obtained from artificial insemination companies. Two different ejaculates of each bull were evaluated with two straws pooled per ejaculate. After thawing (37°C, 30 s), sperm morphology was assessed by staining with Diff-Quik (Siemens Healthcare, Germany). Sperm total motility was investigated immediately after thawing by phase-contrast microscopy using the Leica DM 1000 microscope (Leica, Germany). Viability of spermatozoa was investigated after thawing by mixing 5 µl of the thawed ejaculate with aqueous Eosin Y solution (Sigma Aldrich, Germany) in a volume ratio of 1∶1. Intact and viable spermatozoa stay colourless whereas spermatozoa with disturbed membrane integrity stain red. Counting of viable sperm was done within 10 seconds after mixing. Two hundred spermatozoa in at least two different fields of view were investigated at a magnification of 400× to analyse morphology, viability and motility.

### Assessment of ejaculate volume, sperm count and progressive motility

Fresh semen traits (ejaculate volume, sperm count and progressive motility) of 203 AI bulls (177 *wt/wt*, 21*wt/mt*, 5 *mt/mt*) were provided by an artificial insemination company. Semen quality was analysed based on 10,682 ejaculates with an average of 52.6 ejaculates per bull. At semen collection, the age of the bulls ranged from 1.1 to 2.5 years.

### Immunohistochemical localization of TMEM95

Immunohistochemistry on cryopreserved sperm specimens of 33 bulls (10 *wt/wt*, 10 *wt/mt*,13 *mt/mt*) was repeated for 3 times. After thawing (37°C, 30 s), spermatozoa were washed in phosphate buffered saline (PBS) twice and were diluted in PBS to a concentration of 500,000 spermatozoa/ml. Thereafter, drops of 7 µl were placed on 3-aminopropyl-ethoxysilane-coated slides and dried on a heating-plate at a temperature of 38°C. Subsequently, the slides were fixed in Bouin's solution for 7 min and washed in PBS twice. Non-specific binding was blocked by incubation in blocking buffer (0.1% bovine serum Albumin in PBS, Sigma-Aldrich, Germany) for 5 minutes and in normal goat serum (dilution 1∶5 in PBS, Invitrogen, Germany). Next, the spermatozoa were incubated with the first antibody Yomics Ab989 (mouse-derived against human TMEM95, Primm, USA) in a dilution of 1∶200 in blocking buffer at 4°C overnight. The secondary antibody was the Fluorescein (FITC)-conjugated AffiniPure Goat anti Mouse IgG (H+L) (Dianova, Germany, dilution 1∶200). Negative controls were done by a) replacing the first antibody with PBS and b) using a non-relevant anti-mouse antibody directed against Villin (1∶75, Beckman Coulter). Specimens were evaluated by using a confocal laser scanning microscope (Leica DM IRBE) in magnifications from 400 to 800.

## Supporting Information

Figure S1 Male reproductive ability of 7962 artificial insemination bulls. Male reproductive ability (MRA) in 7962 artificial insemination bulls of the Fleckvieh population (A). Male reproductive ability is highly correlated (r = 0.59) with the 56-day non-return rate in cows (B). Red dots represent 49 bulls with unexplained exceptionally poor reproductive performance ( = subfertile animals).
(TIF)

Figure S2 Genome-wide association study using male reproductive ability as quantitative trait. Association of 652,856 SNPs with male reproductive ability (MRA). P-values were obtained using a mixed-model based GWAS and phenotypes for MRA in 7962 artificial insemination bulls of the FV population.
(TIF)

Figure S3 CNV-analysis within the segment of extended homozygosity. Signal intensities obtained from genotyping with the Illumina BovineHD Bead chip are displayed as log R ratios for cases and controls within the segment of extended homozygosity. The log R ratio is displayed for 3-SNP-sliding windows.
(PNG)

Figure S4 Frequency of the subfertility-associated haplotype in the Fleckvieh population. Genotypes of 19,014 FV bulls used for routine genomic breeding value estimation were analysed. Haplotype analysis revealed an increasing frequency of heterozygous bulls within the last years. In 2009, 23.41% of all genotyped bulls were carrier of the deleterious haplotype. The solid green line represents the carrier frequency as a function of the birth year and the dashed green line is the corresponding regression line (ß = 0.003, P = 0.0002).
(TIF)

Figure S5 Pedigree of 40 homozygous artificial insemination bulls. The pedigree was constructed for 40 homozygous bulls (red) and includes obligate carriers of the haplotype only. Females and males are displayed with ovals and boxes, respectively. Grey colour indicates animals for which genotypes were available. One animal with incomplete pedigree information is displayed in yellow. The blue box represents *HAXL* (*1966), the supposed founder of the mutation. Green asterisks represent re-sequenced animals.
(TIF)

Figure S6 Exploiting whole genome re-sequencing data of 43 animals for the identification of the underlying mutation. Whole genome re-sequencing of 43 animals and subsequent multi-sample variant calling yielded genotypes at 5965 polymorphic sites (5287 SNPs, 678 INDELs) within the 1386 kb segment of extended homozygosity on BTA19. Six of 43 re-sequenced animals were carriers of the subfertility-associated haplotype. One of the carriers was sequenced at high coverage (HC) whereas the remaining five carriers were sequenced at low coverage (LC).
(PDF)

Figure S7 Validation of the nonsense mutation in six heterozygous animals. IGV screen-shots of the nonsense mutation in exon 6 of *TMEM95* (*rs378652941*, c.483C>A, p.Cys161X, Chr19:27689622) for six animals carrying the subfertility-associated haplotype. The mutation was present in the re-sequencing data of four heterozygous animals (C, D, E, F), whereas the mutation could not be identified in the re-sequencing data of two animals (A, B). Sequencing of PCR products revealed that the mutation is present in these animals but initially remained undetected due to the low-coverage sequencing strategy.
(TIF)

Figure S8 Validation of the nonsense mutation in one unaffected animal. The nonsense mutation was identified in the re-sequencing data of one animal not carrying the subfertility-associated haplotype. Sequencing of genomic PCR products revealed that this was a mis-call in the re-sequencing data due to the low coverage sequencing data. The mutation indeed is not present in that animal, as indicated by haplotype analysis.
(TIF)

Figure S9 Topology prediction of transmembrane protein 95. The topology of bovine transmembrane protein 95 (NCBI reference sequence: XP_002695846.1) was predicted with *SPOCTOPUS* (A) and *PHOBIUS* (B). Both methods simultaneously predict N-terminal signal peptide sequences and transmembrane domains. Both tools consistently predicted that transmembrane protein 95 is a single-pass type I transmembrane protein with an extracellular N-terminal signal peptide sequence. The affected codon (p.Cys161X, orange triangle) resides within the predicted transmembrane domain.
(TIF)

Figure S10 Multi-species sequence alignment of transmembrane protein 95. *ClustalW* was used for multiple sequence alignment of the protein sequence of transmembrane protein 95. Red vertical lines indicate the boundaries of different domains predicted with *SPOCTOPUS* and *PHOBIUS*. Protein sequences were obtained from NCBI for *Felis catus* (XP_003996306.1), *Pan troglodytes* (XP_529925.2), *Saimiri boliviensis boliviensis* (XP_003929208.1), *Papio anubis* (XP_003912296.1), *Macaca mulatta* (NP_001181311.1), *Mus musculus* (NP_001182639.1), *Pan paniscus* (XP_003810140.1), *Otolemur garnettii* (XP_003791206.1), *Pongo abeli*i (XP_002827002.1), *Homo sapiens* (NP_937797.1), R*attus norvegicus* (NP_001128271.1), *Callithrix jacchus* (XP_002748028.1), *Canis lupus familiaris* (XP_849662.1) and *Bos taurus* (XP_002695846.1).
(TIF)

Figure S11 Negative control of the primary antibody.
(TIF)

Figure S12 Genome-wide association study for male reproductive ability conditional on the *rs378652941*-polymorphism. The GWAS was repeated using only 7922 animals that are not homozygous for the p.Cys161X-mutation. P-values were obtained using a mixed-model based GWAS.
(TIF)

Figure S13 Polymorphic sites in human *TMEM95*. The genomic structure of the human *TMEM95* (HGNC:27898) gene is shown according to the GRCh37.p10 assembly of the human genome. *TMEM95* consists of seven exons. Known variants within human *TMEM95* were obtained from Ensembl (release 69, October 2012; http://www.ensembl.org). Vertical colored bars indicate the position of known variants within *TMEM95*, among them two stop-gained mutations in exon 1 (rs150578277) and exon 5 (rs199658290), respectively.
(TIF)

Table S1 Gene content within the segment of extended homozygosity on bovine chromosome 19. The gene content was assessed based on the UMD3.1-assembly of the bovine genome sequence. A total of 80 transcripts were identified within the segment of extended homozygosity.
(PDF)

Table S2 Reproductive performance of 40 subfertile animals. Phenotypes for male reproductive ability (MRA) and the 56-day non-return rate are presented for 40 subfertile animals. Inseminations resulting in progeny were considered as successful.
(PDF)

Table S3Effect of the male subfertility-associated haplotype on reproduction and production traits of primiparous cows. Phenotypes in the form of estimated breeding values (EBVs) for six reproduction and three milk production traits were available for 1857 genotyped primiparous cows. The reproduction traits comprise EBVs for overall fertility, EBVs for the 56 non-return rate for heifers (NR56_heifer_) and cows (NR56_cow_), EBVs for the time from calving to first insemination (CTFI) and EBVs for the interval from first to last insemination for heifers (IFL_heifer_) and cows (IFL_cow_). The mean and standard deviation is presented for all 1857 primiparous cows as well as for three groups of animals carrying 0 (unaffected), 1 (carrier) and 2 (homozygous) copies of the subfertility-associated haplotype, respectively. P-values were obtained by fitting the mixed linear model 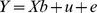, where Y is a vector of phenotypes, b is the effect of the subfertility-associated haplotype, X is a design matrix of haplotype genotypes coded as 0, 1 and 2, respectively, u is the polygenic term ∼N(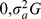), with 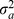 being the additive genetic variance and G is the realized genomic relationship matrix (GRM) among 1857 primiparous cows built based on 635,224 autosomal SNPs and e is a vector of random residual effects.(PDF)

Table S4 Single nucleotide polymorphisms on BTA19 compatible with the supposed recessive inheritance pattern. The mutation scan revealed 26 SNPs fulfilling three criteria required to be compatible with the supposed recessive inheritance. Genotypes are presented separately for six animals carrying the subfertility-associated haplotype and for 37 animals not carrying the subfertility-associated haplotype. Observed genotypes are displayed as homozygous for the reference allele (HOM_ref), heterozygous (HET) and homozygous for the non-reference allele (HOM_alt).
(PDF)

Table S5 Insertion/Deletion-polymorphisms on BTA19 compatible to the recessive inheritance pattern. The mutation scan revealed six INDELs fulfilling three criteria required to be compatible with the supposed recessive inheritance. Genotypes are presented separately for six animals carrying the subfertility-associated haplotype and for 37 animals not carrying the subfertility-associated haplotype. Observed genotypes are displayed as homozygous for the reference allele (HOM_ref), heterozygous (HET) and homozygous for the non-reference allele (HOM_alt).
(PDF)

Table S6 Reproductive performance of nine subfertile animals not attributable to the c.483C>A - mutation. Inseminations resulting in progeny were considered as successful.
(PDF)

Table S7 Primers and probes used for TaqMan genotyping assays.
(PDF)
