# Supplementary figures and images for: A Nonsense Mutation in TMEM95 Encoding a Nondescript Transmembrane Protein Causes Idiopathic Male Subfertility in Cattle
Source: PLoS Genet. 2014 Jan 2;10(1):e1004044. doi: 10.1371/journal.pgen.1004044 (PMC3879157; doi:10.1371/journal.pgen.1004044)

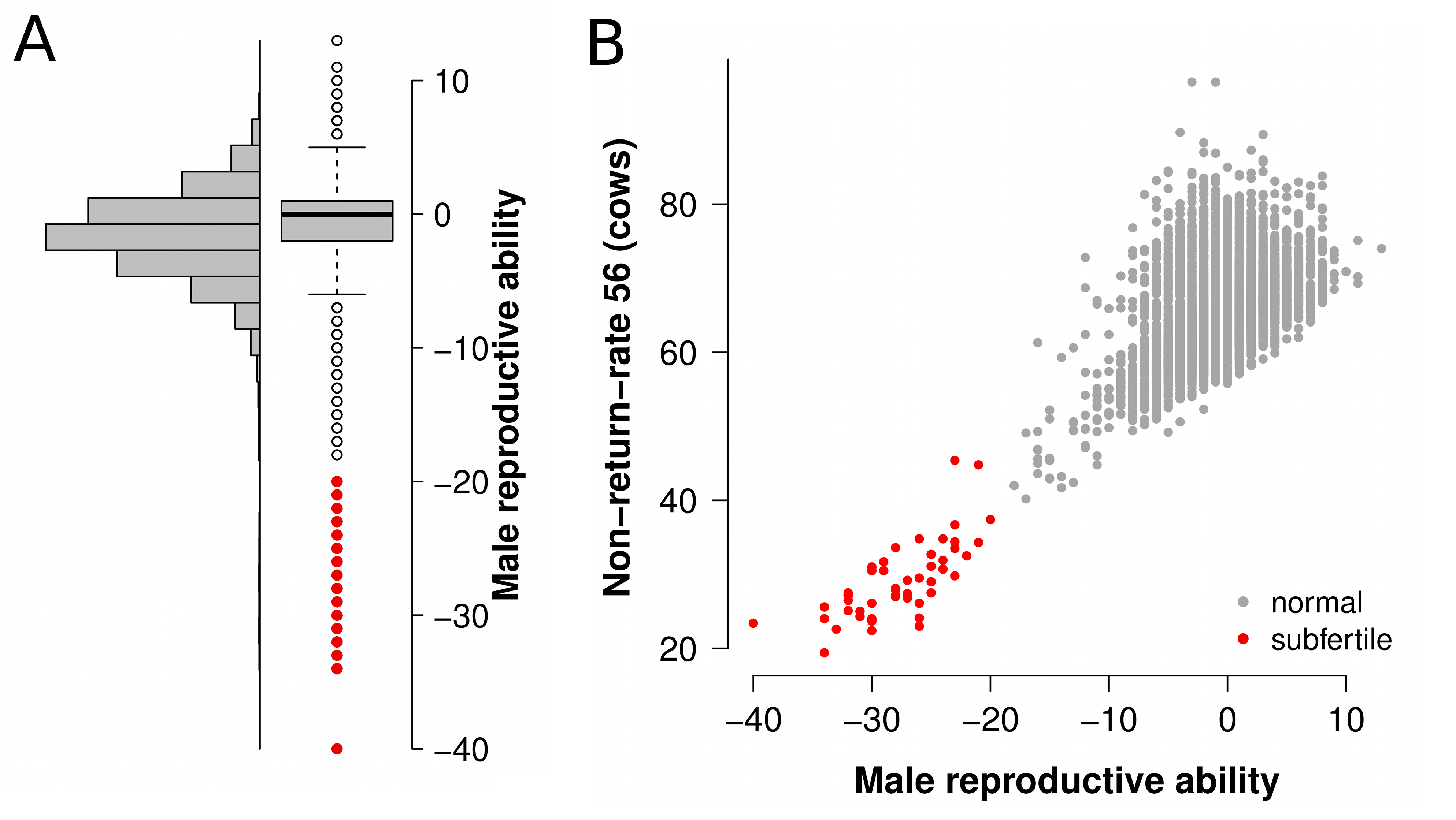

Supplement: Figure S1 — Male reproductive ability of 7962 artificial insemination bulls. Male reproductive ability (MRA) in 7962 artificial insemination bulls of the Fleckvieh population (A). Male reproductive ability is highly correlated (r = 0.59) with the 56-day non-return rate in cows (B). Red dots represent 49 bulls with unexplained exceptionally poor reproductive performance ( = subfertile animals). (TIF) [file pgen.1004044.s001.tif]

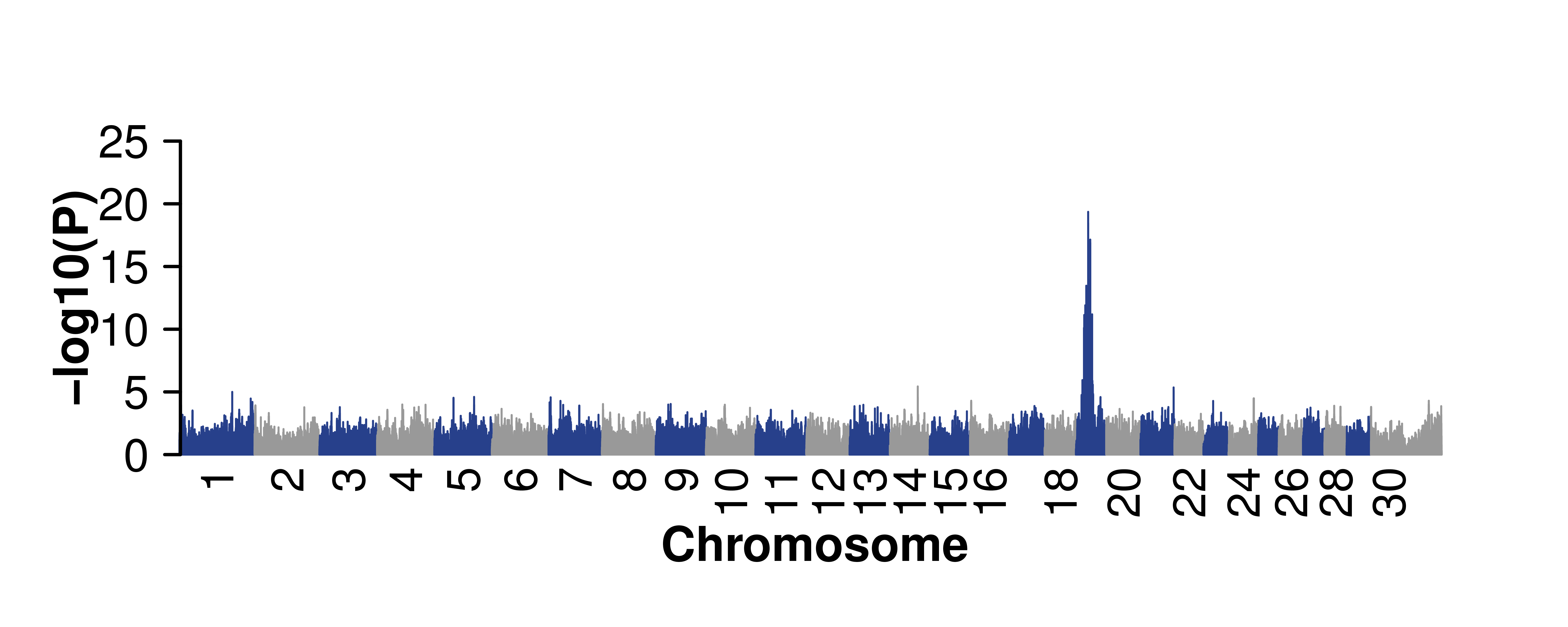

Supplement: Figure S2 — Genome-wide association study using male reproductive ability as quantitative trait. Association of 652,856 SNPs with male reproductive ability (MRA). P-values were obtained using a mixed-model based GWAS and phenotypes for MRA in 7962 artificial insemination bulls of the FV population. (TIF) [file pgen.1004044.s002.tif]

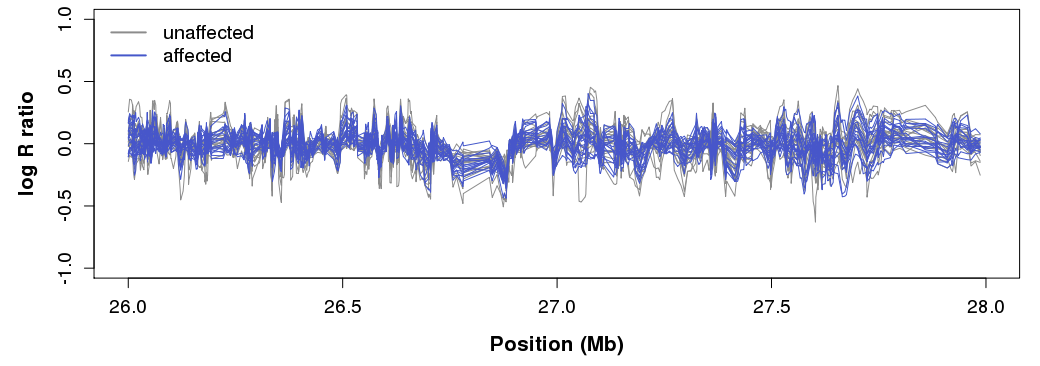

Supplement: Figure S3 — CNV-analysis within the segment of extended homozygosity. Signal intensities obtained from genotyping with the Illumina BovineHD Bead chip are displayed as log R ratios for cases and controls within the segment of extended homozygosity. The log R ratio is displayed for 3-SNP-sliding windows. (PNG) [file pgen.1004044.s003.png]

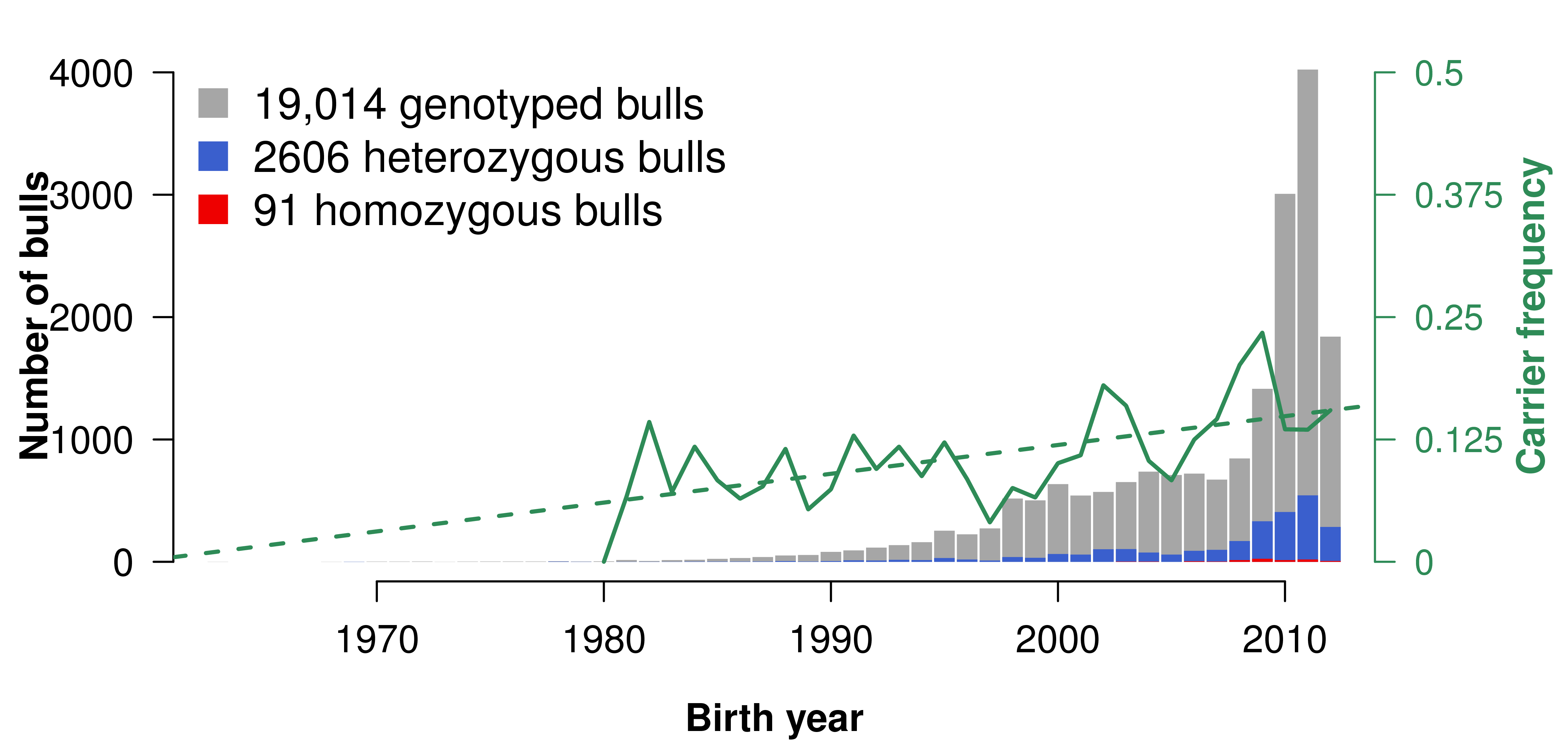

Supplement: Figure S4 — Frequency of the subfertility-associated haplotype in the Fleckvieh population. Genotypes of 19,014 FV bulls used for routine genomic breeding value estimation were analysed. Haplotype analysis revealed an increasing frequency of heterozygous bulls within the last years. In 2009, 23.41% of all genotyped bulls were carrier of the deleterious haplotype. The solid green line represents the carrier frequency as a function of the birth year and the dashed green line is the corresponding regression line (ß = 0.003, P = 0.0002). (TIF) [file pgen.1004044.s004.tif]

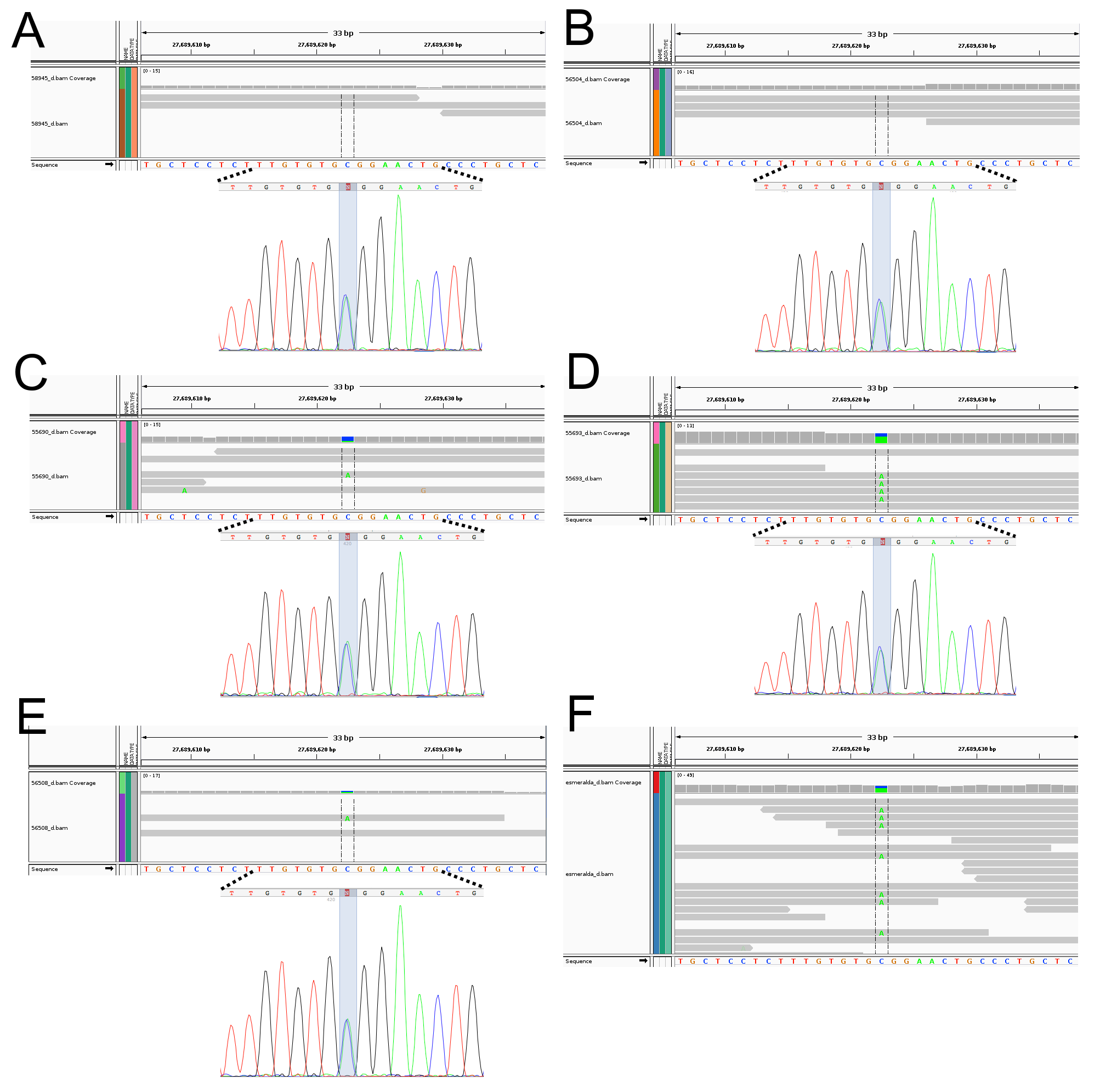

Supplement: Figure S7 — Validation of the nonsense mutation in six heterozygous animals. IGV screen-shots of the nonsense mutation in exon 6 of TMEM95 (rs378652941, c.483C>A, p.Cys161X, Chr19:27689622) for six animals carrying the subfertility-associated haplotype. The mutation was present in the re-sequencing data of four heterozygous animals (C, D, E, F), whereas the mutation could not be identified in the re-sequencing data of two animals (A, B). Sequencing of PCR products revealed that the mutation is present in these animals but initially remained undetected due to the low-coverage sequencing strategy. (TIF) [file pgen.1004044.s007.tif]

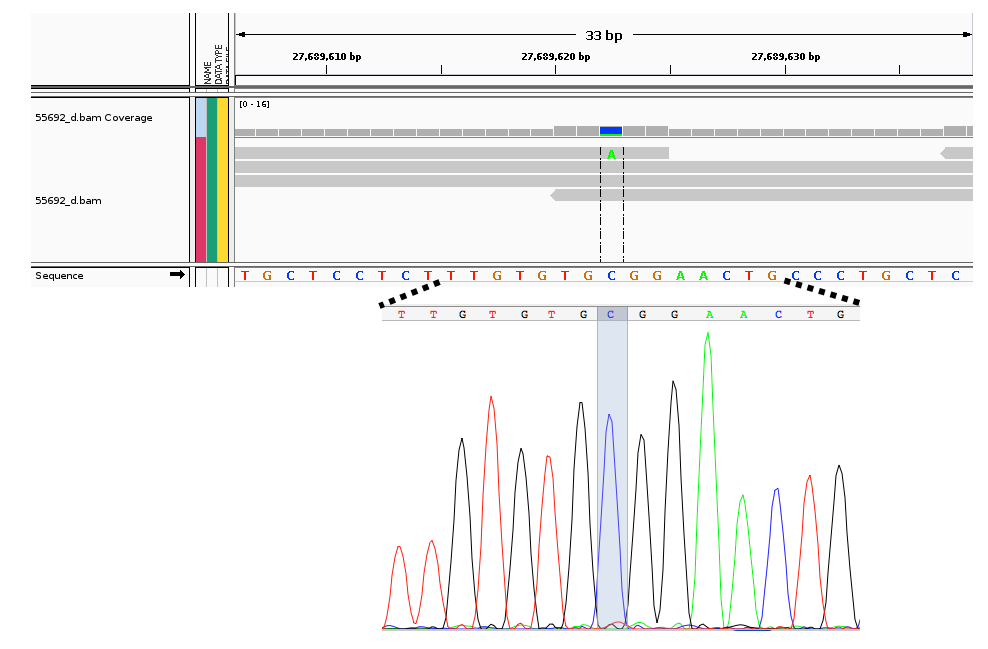

Supplement: Figure S8 — Validation of the nonsense mutation in one unaffected animal. The nonsense mutation was identified in the re-sequencing data of one animal not carrying the subfertility-associated haplotype. Sequencing of genomic PCR products revealed that this was a mis-call in the re-sequencing data due to the low coverage sequencing data. The mutation indeed is not present in that animal, as indicated by haplotype analysis. (TIF) [file pgen.1004044.s008.tif]

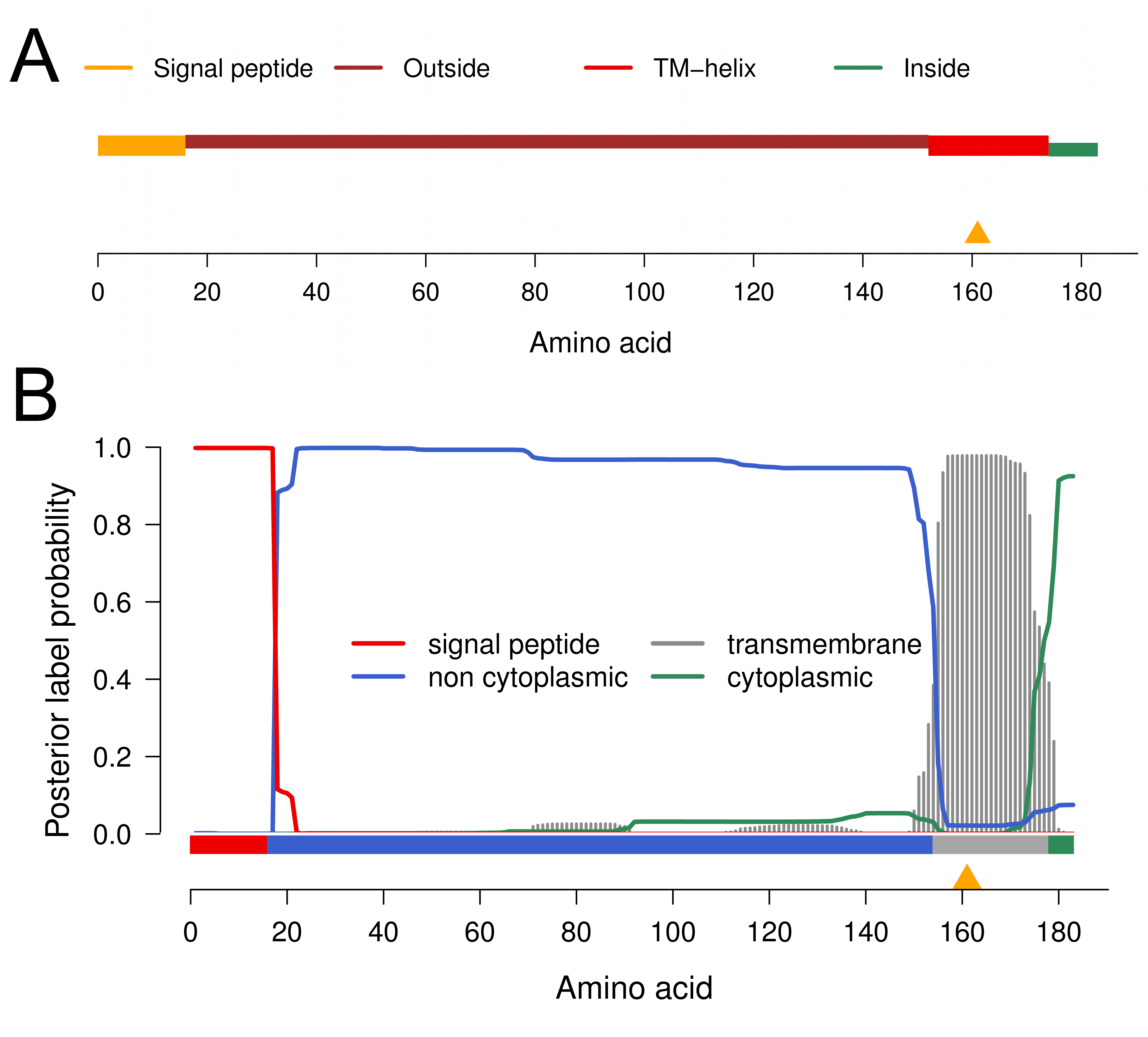

Supplement: Figure S9 — Topology prediction of transmembrane protein 95. The topology of bovine transmembrane protein 95 (NCBI reference sequence: XP_002695846.1) was predicted with SPOCTOPUS (A) and PHOBIUS (B). Both methods simultaneously predict N-terminal signal peptide sequences and transmembrane domains. Both tools consistently predicted that transmembrane protein 95 is a single-pass type I transmembrane protein with an extracellular N-terminal signal peptide sequence. The affected codon (p.Cys161X, orange triangle) resides within the predicted transmembrane domain. (TIF) [file pgen.1004044.s009.tif]

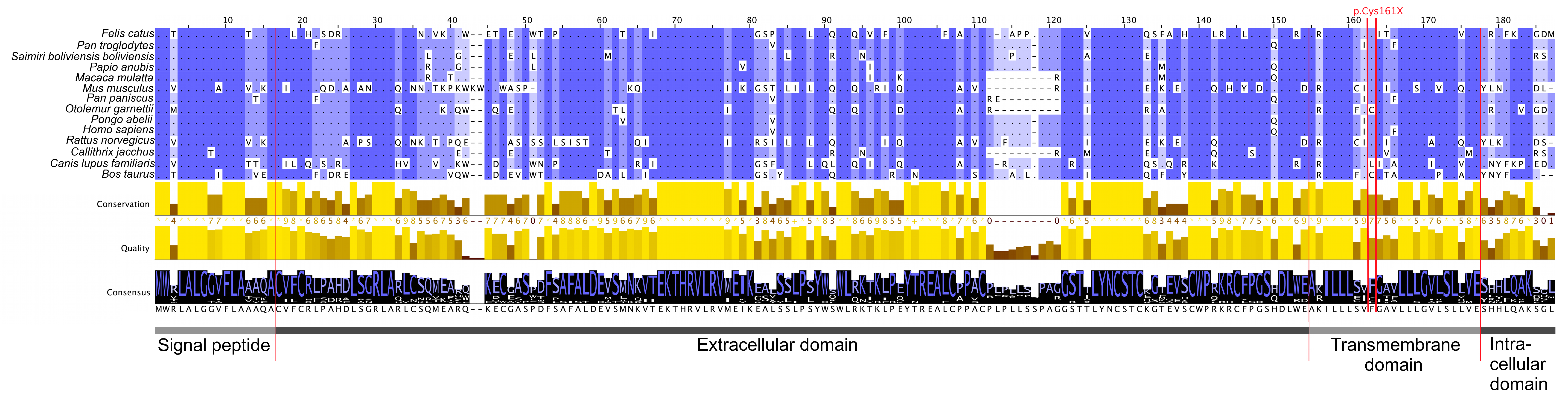

Supplement: Figure S10 — Multi-species sequence alignment of transmembrane protein 95. ClustalW was used for multiple sequence alignment of the protein sequence of transmembrane protein 95. Red vertical lines indicate the boundaries of different domains predicted with SPOCTOPUS and PHOBIUS. Protein sequences were obtained from NCBI for Felis catus (XP_003996306.1), Pan troglodytes (XP_529925.2), Saimiri boliviensis boliviensis (XP_003929208.1), Papio anubis (XP_003912296.1), Macaca mulatta (NP_001181311.1), Mus musculus (NP_001182639.1), Pan paniscus (XP_003810140.1), Otolemur garnettii (XP_003791206.1), Pongo abelii (XP_002827002.1), Homo sapiens (NP_937797.1), Rattus norvegicus (NP_001128271.1), Callithrix jacchus (XP_002748028.1), Canis lupus familiaris (XP_849662.1) and Bos taurus (XP_002695846.1). (TIF) [file pgen.1004044.s010.tif]

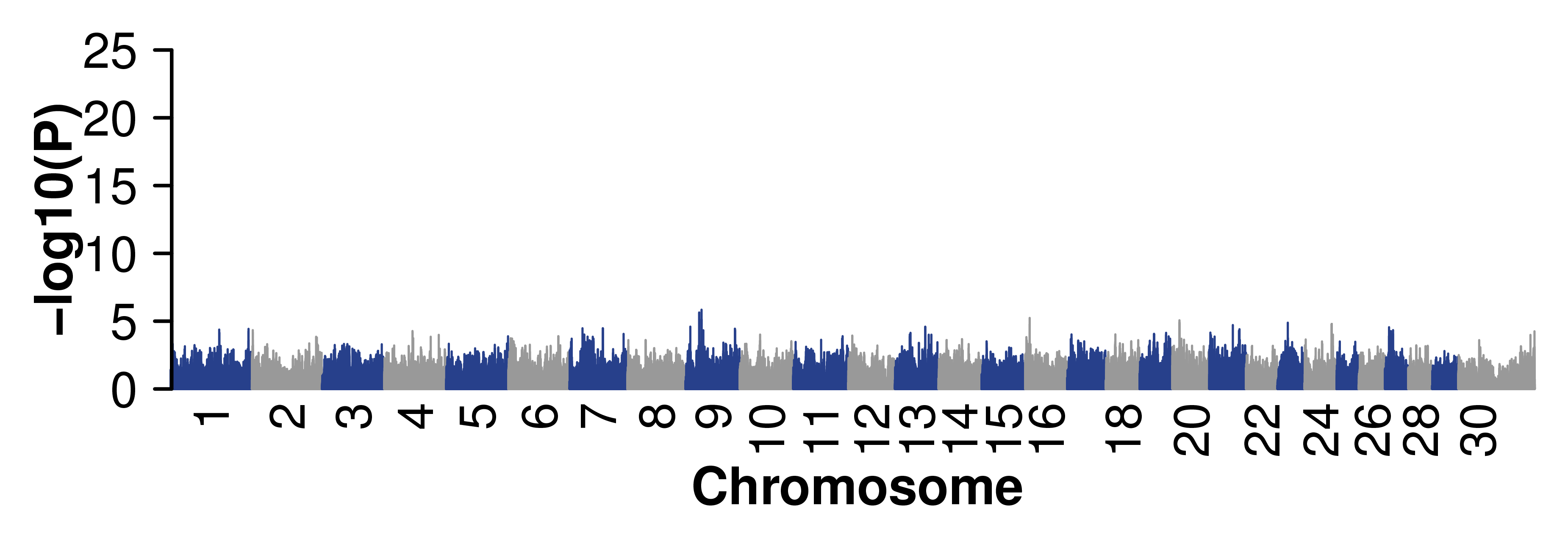

Supplement: Figure S12 — Genome-wide association study for male reproductive ability conditional on the rs378652941-polymorphism. The GWAS was repeated using only 7922 animals that are not homozygous for the p.Cys161X-mutation. P-values were obtained using a mixed-model based GWAS. (TIF) [file pgen.1004044.s012.tif]

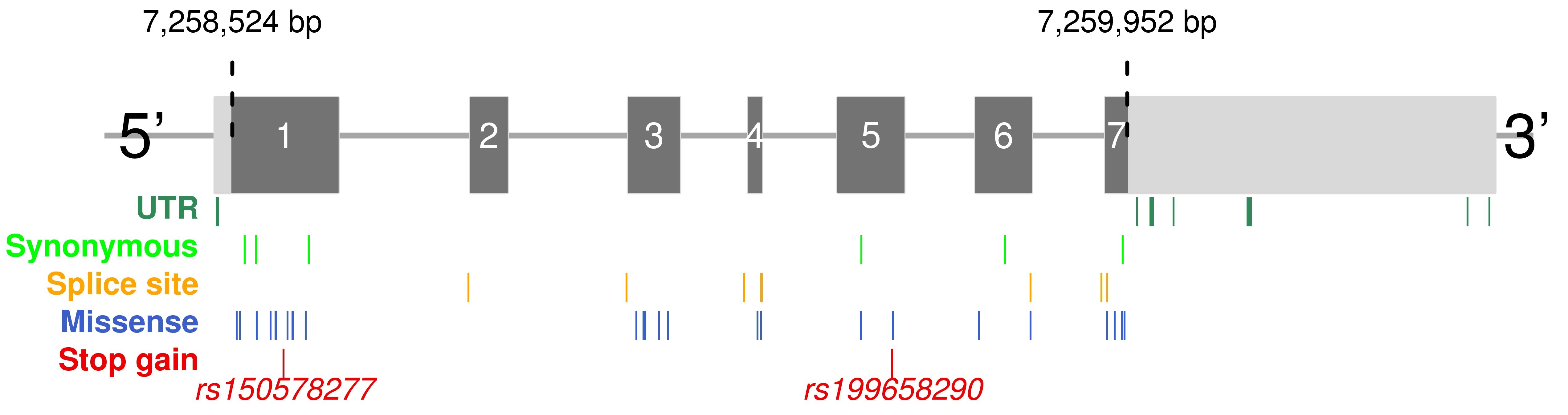

Supplement: Figure S13 — Polymorphic sites in human TMEM95. The genomic structure of the human TMEM95 (HGNC:27898) gene is shown according to the GRCh37.p10 assembly of the human genome. TMEM95 consists of seven exons. Known variants within human TMEM95 were obtained from Ensembl (release 69, October 2012; http://www.ensembl.org). Vertical colored bars indicate the position of known variants within TMEM95, among them two stop-gained mutations in exon 1 (rs150578277) and exon 5 (rs199658290), respectively. (TIF) [file pgen.1004044.s013.tif]
