# Supplementary material for: A Nonsense Mutation in TMEM95 Encoding a Nondescript Transmembrane Protein Causes Idiopathic Male Subfertility in Cattle
Source: PLoS Genet. 2014 Jan 2;10(1):e1004044. doi: 10.1371/journal.pgen.1004044 (PMC3879157; doi:10.1371/journal.pgen.1004044)
Supplement: Figure S6 — Exploiting whole genome re-sequencing data of 43 animals for the identification of the underlying mutation. Whole genome re-sequencing of 43 animals and subsequent multi-sample variant calling yielded genotypes at 5965 polymorphic sites (5287 SNPs, 678 INDELs) within the 1386 kb segment of extended homozygosity on BTA19. Six of 43 re-sequenced animals were carriers of the subfertility-associated haplotype. One of the carriers was sequenced at high coverage (HC) whereas the remaining five carriers were sequenced at low coverage (LC). (PDF) [file pgen.1004044.s006.pdf]

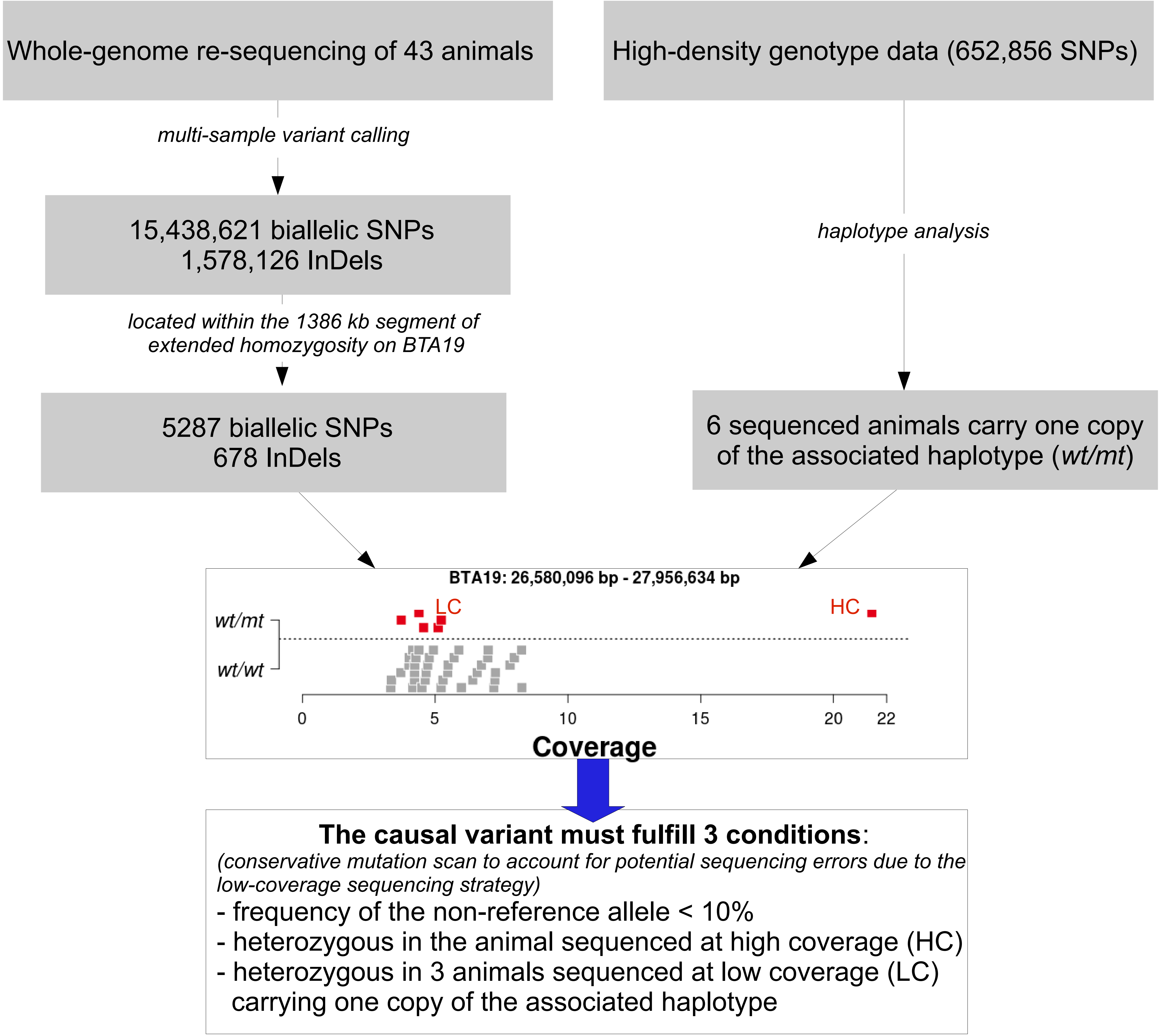
Whole-genome re-sequencing of 43 animals
multi-sample variant calling
15,438,621 biallelic SNPs
1,578,126 InDels
located within the 1386 kb segment of extended homozygosity on BTA19
5287 biallelic SNPs
678 InDels
High-density genotype data (652,856 SNPs)
haplotype analysis
6 sequenced animals carry one copy of the associated haplotype (wt/mt)
BTA19: 26,580,096 bp - 27,956,634 bp
LC
HC
wt/mt
wt/wt
0
5
10
15
20
22
Coverage
The causal variant must fulfill 3 conditions:
(conservative mutation scan to account for potential sequencing errors due to the low-coverage sequencing strategy)
- frequency of the non-reference allele < 10%
- heterozygous in the animal sequenced at high coverage (HC)
- heterozygous in 3 animals sequenced at low coverage (LC) carrying one copy of the associated haplotype
