# Supplementary material for: A Nonsense Mutation in TMEM95 Encoding a Nondescript Transmembrane Protein Causes Idiopathic Male Subfertility in Cattle
Source: PLoS Genet. 2014 Jan 2;10(1):e1004044. doi: 10.1371/journal.pgen.1004044 (PMC3879157; doi:10.1371/journal.pgen.1004044)
Supplement: Table S1 — Gene content within the segment of extended homozygosity on bovine chromosome 19. The gene content was assessed based on the UMD3.1-assembly of the bovine genome sequence. A total of 80 transcripts were identified within the segment of extended homozygosity. (PDF) [file pgen.1004044.s014.pdf]

Gene		Start of translation	Stop of translation	Strand
Symbol	Full name			
LOC528166	NLR family, pyrin domain containing 1	26,638,785	26,667,746	+
LOC788205	uncharacterized LOC788205	26,675,245	26,676,864	+
MIS12	MIND kinetochore complex component, homolog (S. pombe)	26,682,795	26,678,016	-
DERL2	Der1-like domain family, member 2	26,683,201	26,691,726	+
DHX33	DEAH (Asp-Glu-Ala-His) box polypeptide 33	26,702,292	26,715,381	+
C1QBP	complement component 1, q subcomponent binding protein	26,724,673	26,730,101	+
RPAIN	RPA interacting protein	26,737,179	26,730,162	-
NUP88	nucleoporin 88kDa	26,738,050	26,759,721	+
RABEP1	rabaptin, RAB GTPase binding effector protein 1	26,870,523	26,761,840	-
ZFP3	zinc finger protein 3 homolog (mouse)	26,975,114	26,968,016	-
KIF1C	kinesin family member 1C	27,043,780	27,013,896	-
SPAG7	sperm associated antigen 7	27,068,573	27,072,720	+
ENO3	enolase 3 (beta, muscle)	27,078,655	27,073,496	-
PFN1	profilin 1	27,081,319	27,084,643	+
RNF167	ring finger protein 167	27,087,301	27,085,162	-
SLC25A11	solute carrier family 25 (mitochondrial carrier; oxoglutarate carrier), member 11	27,089,299	27,092,026	+
GP1BA	glycoprotein Ib (platelet), alpha polypeptide	27,096,509	27,093,800	-
CHRNE	cholinergic receptor, nicotinic, epsilon (muscle)	27,118,517	27,123,114	+
LOC100137824	chromosome 19 open reading frame, human C17orf107	27,121,973	27,121,211	-
MINK1	misshapen-like kinase 1	27,170,653	27,123,625	-
PLD2	phospholipase D2	27,189,861	27,176,940	-
PSMB6	proteasome (prosome, macropain) subunit, beta type, 6	27,200,805	27,198,592	-
LOC100300004	glycolipid transfer protein domain containing 2	27,204,709	27,206,013	+
LOC526730	vitelline membrane outer layer 1 homolog (chicken)	27,208,472	27,209,491	+
TM4SF5	transmembrane 4 L six family member 5	27,216,974	27,210,645	-
ZMYND15	zinc finger, MYND-type containing 15	27,248,391	27,243,294	-
CXCL16	chemokine (C-X-C motif) ligand 16	27,249,528	27,253,097	+
MED11	mediator complex subunit 11	27,255,303	27,253,467	-
ARRB2	arrestin, beta 2	27,277,055	27,268,294	-
PELP1	proline, glutamate and leucine rich protein 1	27,282,084	27,303,568	+
ALOX15	arachidonate 15-lipoxygenase	27,331,077	27,339,653	+
ALOX12E	arachidonate lipoxygenase, epidermal	27,361,427	27,370,259	+
ALOX12	arachidonate 12-lipoxygenase	27,421,125	27,434,258	+
RNASEK	ribonuclease, RNase A family, 1 (pancreatic)	27,436,503	27,438,228	+
C19H17orf49	chromosome 19 open reading frame, human C17orf49	27,438,490	27,441,265	+
BCL6B	B-cell CLL/lymphoma 6, member B	27,447,122	27,451,108	+
SLC16A13	solute carrier family 16, member 13 (monocarboxylic acid transporter 13)	27,455,597	27,459,089	+
SLC16A11	solute carrier family 16, member 11 (monocarboxylic acid transporter 11)	27,461,757	27,459,401	-
LOC514833	C-type lectin domain family 10, member A	27,469,941	27,467,160	-
ASGR2	asialoglycoprotein receptor 2	27,500,094	27,490,117	-
ASGR1	asialoglycoprotein receptor 1	27,532,634	27,529,097	-
DLG4	discs, large homolog 4 (Drosophila)	27,565,410	27,543,283	-
ACADVL	acyl-CoA dehydrogenase, very long chain	27,568,181	27,573,378	+

DVL2	dishevelled, dsh homolog 2 (Drosophila)	27,581,405	27,573,452	-
PHF23	PHD finger protein 23	27,585,939	27,582,170	-
GABARAP	GABA(A) receptor-associated protein	27,588,815	27,586,884	-
CTDNEP1	CTD nuclear envelope phosphatase 1	27,596,032	27,589,893	-
ELP5	elongator acetyltransferase complex subunit 5	27,596,732	27,602,468	+
CLDN7	claudin 7	27,604,817	27,602,592	-
SLC2A4	solute carrier family 2 (facilitated glucose transporter), member 4	27,616,613	27,622,203	+
YBX2	Y box binding protein 2	27,628,780	27,622,773	-
EIF5A	eukaryotic translation initiation factor 5A	27,648,382	27,653,258	+
GPS2	G protein pathway suppressor 2	27,656,067	27,653,444	-
NEURL4	neuralized homolog 4 (Drosophila)	27,668,154	27,656,328	-
LOC508096	protein phosphatase inhibitor 2-like	27,669,268	27,672,493	+
ACAP1	ArfGAP with coiled-coil, ankyrin repeat and PH domains 1	27,673,004	27,684,649	+
KCTD11	potassium channel tetramerisation domain containing 11	27,685,379	27,688,137	+
TMEM95	**transmembrane protein 95**	**27,688,399**	**27,689,818**	**+**
TNK1	tyrosine kinase, non-receptor, 1	27,698,156	27,706,416	+
PLSCR3	phospholipid scramblase 3	27,710,913	27,706,379	-
C19H17orf61	chromosome 19 open reading frame, human C17orf61	27,715,619	27,714,469	-
LOC100337038	neuroligin-2-like	27,727,210	27,722,492	-
NLGN2	neuroligin 2	27,727,805	27,733,393	+
SPEM1	spermatid maturation 1	27,733,908	27,735,192	+
TMEM102	transmembrane protein 102	27,747,547	27,749,622	+
FGF11	fibroblast growth factor 11	27,751,729	27,755,716	+
CHRNB1	cholinergic receptor, nicotinic, beta 1 (muscle)	27,757,183	27,766,382	+
ZBTB4	zinc finger and BTB domain containing 4	27,773,342	27,767,024	-
AMAC1L3	acyl-malonyl condensing enzyme 1-like 3	27,784,052	27,785,626	+
POLR2A	polymerase (RNA) II (DNA directed) polypeptide A, 220kDa	27,786,959	27,803,965	+
LOC100337330	DNA-directed RNA polymerase II A-like	27,812,287	27,813,659	+
TNFSF13	tumor necrosis factor (ligand) superfamily, member 13	27,901,945	27,904,805	+
SENP3	SUMO1/sentrin/SMT3 specific peptidase 3	27,906,586	27,914,530	+
EIF4A1	eukaryotic translation initiation factor 4A1	27,915,413	27,921,421	+
CD68	CD68 molecule	27,921,927	27,923,983	+
MPDU1	mannose-P-dolichol utilization defect 1	27,925,701	27,929,096	+
SOX15	SRY (sex determining region Y)-box 15	27,930,911	27,929,104	-
FXR2	fragile X mental retardation, autosomal homolog 2	27,945,443	27,932,223	-
SAT2	spermidine/spermine N1-acetyltransferase family member 2	27,955,396	27,953,738	-
SHBG	sex hormone-binding globulin	27,955,573	27,960,099	+
