# Supplementary material for: A Nonsense Mutation in TMEM95 Encoding a Nondescript Transmembrane Protein Causes Idiopathic Male Subfertility in Cattle
Source: PLoS Genet. 2014 Jan 2;10(1):e1004044. doi: 10.1371/journal.pgen.1004044 (PMC3879157; doi:10.1371/journal.pgen.1004044)
Supplement: Table S2 — Reproductive performance of 40 subfertile animals. Phenotypes for male reproductive ability (MRA) and the 56-day non-return rate are presented for 40 subfertile animals. Inseminations resulting in progeny were considered as successful. (PDF) [file pgen.1004044.s015.pdf]

Animal ID	Birth year	Male reproductive ability	Non-return-rate 56 (cows) (%)	Number of inseminations	Number of successful inseminations	Proportion of successful inseminations
Subfertile 1	2009	-30	31.0	1709	27	0.0158
Subfertile 2	2003	-40	23.4	633	13	0.0205
Subfertile 3	2004	-29	30.5	819	11	0.0134
Subfertile 4	2004	-32	25.1	1046	30	0.0287
Subfertile 5	2003	-32	27.1	1344	15	0.0112
Subfertile 6	2008	-32	27.5	1236	28	0.0227
Subfertile 7	2008	-24	30.7	662	9	0.0136
Subfertile 8	2008	-27	27.4	1207	56	0.0464
Subfertile 9	2009	-26	26.1	1003	34	0.0339
Subfertile 10	2006	-32	27.2	610	28	0.0459
Subfertile 11	2007	-34	24.0	832	8	0.0096
Subfertile 12	2006	-31	25.0	665	11	0.0165
Subfertile 13	2006	-28	27.2	1486	21	0.0141
Subfertile 14	2006	-28	33.6	816	11	0.0135
Subfertile 15	2006	-25	31.1	811	14	0.0173
Subfertile 16	2007	-23	34.4	1267	24	0.0189
Subfertile 17	2008	-25	29.0	921	16	0.0174
Subfertile 18	2007	-33	22.6	1103	9	0.0082
Subfertile 19	2008	-30	22.4	666	5	0.0075
Subfertile 20	2008	-29	31.7	846	10	0.0118
Subfertile 21	2008	-23	36.7	717	8	0.0112
Subfertile 22	2009	-34	25.6	719	14	0.0195
Subfertile 23	2009	-28	28.1	849	14	0.0165
Subfertile 24	2009	-28	27.9	1241	36	0.0290
Subfertile 25	2008	-32	26.5	777	5	0.0064
Subfertile 26	2008	-25	27.5	864	8	0.0093
Subfertile 27	2008	-30	30.5	699	4	0.0057
Subfertile 28	2009	-30	23.7	728	8	0.0110
Subfertile 29	2008	-26	29.5	887	17	0.0192
Subfertile 30	2008	-31	24.3	708	11	0.0155
Subfertile 31	2009	-26	23.0	698	9	0.0129
Subfertile 32	2008	-30	26.1	636	7	0.0110
Subfertile 33	2009	-26	24.1	692	18	0.0260
Subfertile 34	2009	-27	29.2	986	23	0.0233
Subfertile 35	2009	-25	32.7	671	9	0.0134
Subfertile 36	2009	-23	28.8	863	13	0.0151
Subfertile 37	2009	-34	19.4	793	9	0.0113
Subfertile 38	2009	-22	32.5	754	4	0.0053
Subfertile 39	2009	-30	24.0	812	9	0.0111
Subfertile 40	2008	-27	26.8	895	13	0.0145
Ø 40 subfertile animals		-28.67	27.63	891.8	15.5	0.0174
Ø FV population		--	--	--	--	0.4269
