# Supplementary material for: A Nonsense Mutation in TMEM95 Encoding a Nondescript Transmembrane Protein Causes Idiopathic Male Subfertility in Cattle
Source: PLoS Genet. 2014 Jan 2;10(1):e1004044. doi: 10.1371/journal.pgen.1004044 (PMC3879157; doi:10.1371/journal.pgen.1004044)
Supplement: Table S3 — Effect of the male subfertility-associated haplotype on reproduction and production traits of primiparous cows. Phenotypes in the form of estimated breeding values (EBVs) for six reproduction and three milk production traits were available for 1857 genotyped primiparous cows. The reproduction traits comprise EBVs for overall fertility, EBVs for the 56 non-return rate for heifers (NR56heifer) and cows (NR56cow), EBVs for the time from calving to first insemination (CTFI) and EBVs for the interval from first to last insemination for heifers (IFLheifer) and cows (IFLcow). The mean and standard deviation is presented for all 1857 primiparous cows as well as for three groups of animals carrying 0 (unaffected), 1 (carrier) and 2 (homozygous) copies of the subfertility-associated haplotype, respectively. P-values were obtained by fitting the mixed linear model , where Y is a vector of phenotypes, b is the effect of the subfertility-associated haplotype, X is a design matrix of haplotype genotypes coded as 0, 1 and 2, respectively, u is the polygenic term ∼N(), with being the additive genetic variance and G is the realized genomic relationship matrix (GRM) among 1857 primiparous cows built based on 635,224 autosomal SNPs and e is a vector of random residual effects. (PDF) [file pgen.1004044.s016.pdf]

	N	Reproduction traits						Production traits (1st lactation)		
		Overall fertility	$NR56_{heifer}$	$NR56_{cow}$	CTFI	IFL_{heifer}	IFL_{cow}	Milk yield	Fat yield	Protein yield
females	1857	100.51±4.06	98.79±5.24	100.76±4.89	102.03±6.54	99.32±5.81	101.04±5.26	271.1±328.03	5.93±10.98	7.94±9.05
unaffected	1563	100.60±4.08	99.01±5.15	100.89±4.92	102.01±6.51	99.41±5.95	101.05±5.28	273.64±329.71	5.95±11.03	8.14±9.14
carrier	278	99.98±3.95	97.51±5.62	100.05±4.69	102.08±6.73	98.73±5.01	100.94±5.24	261.21±310.34	5.86±10.87	7.04±8.30
homozygous	16	100.81±3.21	99.12±3.69	100.06±3.79	103.19±6.55	101.25±4.70	101.75±5.00	194.44±455.06	5.98±8.56	4.03±11.25
P		0.74	0.38	0.60	0.92	0.34	0.43	0.15	0.72	0.24
