# Supplementary material for: A Nonsense Mutation in TMEM95 Encoding a Nondescript Transmembrane Protein Causes Idiopathic Male Subfertility in Cattle
Source: PLoS Genet. 2014 Jan 2;10(1):e1004044. doi: 10.1371/journal.pgen.1004044 (PMC3879157; doi:10.1371/journal.pgen.1004044)
Supplement: Table S4 — Single nucleotide polymorphisms on BTA19 compatible with the supposed recessive inheritance pattern. The mutation scan revealed 26 SNPs fulfilling three criteria required to be compatible with the supposed recessive inheritance. Genotypes are presented separately for six animals carrying the subfertility-associated haplotype and for 37 animals not carrying the subfertility-associated haplotype. Observed genotypes are displayed as homozygous for the reference allele (HOM_ref), heterozygous (HET) and homozygous for the non-reference allele (HOM_alt). (PDF) [file pgen.1004044.s017.pdf]

Chromosomal Position (UMD3.1)	NCBI rsSNP ID	Refernce allele	6 heterozygous animals			37 controls			Affected Gene	Effect
			HOM_ref	HET	HOM_alt	HOM_ref	HET	HOM_alt		
26,602,288	rs209822541	C	-	6	-	36	1	-	intergenic	
26,699,199	rs208522637	C	-	6	-	37	-	-	intergenic	
26,902,815	rs378821064	C	-	6	-	37	-	-	intergenic	
26,904,203	rs380583481	G	-	6	-	37	-	-	intergenic	
27,042,848	rs381722524	T	-	5	1	36	1	-	*KIF1C*	p.Gln66Arg
27,201,512	rs384157535	T	-	5	1	36	1	-	intergenic	
27,299,006	rs208952900	C	2	4	-	34	2	1	*PELP1*	p.Phe250Phe
27,466,084	rs382969975	G	-	5	1	37	-	-	intergenic	
27,478,464	rs379310683	G	-	6	-	35	2	-	intergenic	
27,479,750	rs380154164	G	1	5	-	35	2	-	intergenic	
27,487,918	rs378958467	G	-	6	-	36	1	-	intergenic	
27,503,656	rs381411314	T	-	6	-	35	2	-	intergenic	
27,511,896	rs385196225	A	-	6	-	35	2	-	intergenic	
27,514,326	rs382243219	G	-	6	-	35	2	-	intergenic	
27,515,879	rs385187938	G	-	6	-	37	-	-	intergenic	
27,570,146	rs385135118	C	2	4	-	36	1	-	*ACADVL*	p.Pro236Thr
27,575,087	rs380843176	C	1	5	-	36	1	-	*DVL2*	Intron 13
27,644,286	rs208400072	G	-	6	-	37	-	-	intergenic	
27,644,896	rs384649516	A	-	6	-	37	-	-	intergenic	
27,649,261	rs384634002	C	1	5	-	37	-	-	*EIF5A*	5'UTR
27,656,771	rs384529202	G	1	5	-	37	-	-	*NEURL4*	3'UTR
27,689,622	rs378652941	C	2	4	-	36	1	-	*TMEM95*	p.Cys161X
27,851,157	rs382014470	C	1	5	-	37	-	-	intergenic	
27,889,622	rs383841120	T	2	4	-	37	-	-	intergenic	
27,915,586	rs383592590	C	-	6	-	35	2	-	*EIF4A1*	Intron 1
27,953,266	rs382682680	G	2	4	-	37	-	-	intergenic	
