# Supplementary material for: A Nonsense Mutation in TMEM95 Encoding a Nondescript Transmembrane Protein Causes Idiopathic Male Subfertility in Cattle
Source: PLoS Genet. 2014 Jan 2;10(1):e1004044. doi: 10.1371/journal.pgen.1004044 (PMC3879157; doi:10.1371/journal.pgen.1004044)
Supplement: Table S5 — Insertion/Deletion-polymorphisms on BTA19 compatible to the recessive inheritance pattern. The mutation scan revealed six INDELs fulfilling three criteria required to be compatible with the supposed recessive inheritance. Genotypes are presented separately for six animals carrying the subfertility-associated haplotype and for 37 animals not carrying the subfertility-associated haplotype. Observed genotypes are displayed as homozygous for the reference allele (HOM_ref), heterozygous (HET) and homozygous for the non-reference allele (HOM_alt). (PDF) [file pgen.1004044.s018.pdf]

Chromosomal Position (UMD3.1)	NCBI rsSNP ID	Refernce sequence	alternative sequence	6 heterozygous animals			37 controls			Affected gene
				HOM_ref	HET	HOM_alt	HOM_ref	HET	HOM_alt	
27,513,219	ss699732590	ACTCT	ACT	-	6	-	35	2	-	intergenic
27,645,080	rs382811517	TCCC	TCC	-	6	-	37	-	-	intergenic
27,645,956	ss699733579	ATTAC	ATTACTTAC	1	5	-	37	-	-	intergenic
27,646,309	rs385981947	AAT	AATCAT	-	6	-	37	-	-	intergenic
27,646,432	rs381059253	AGTTT	AGTTTGTTT	1	5	-	37	-	-	intergenic
27,909,585	rs382596441	CTTT	CTT	1	5	-	37	-	-	*SENP3* (intronic)
