# Supplementary material for: A Nonsense Mutation in TMEM95 Encoding a Nondescript Transmembrane Protein Causes Idiopathic Male Subfertility in Cattle
Source: PLoS Genet. 2014 Jan 2;10(1):e1004044. doi: 10.1371/journal.pgen.1004044 (PMC3879157; doi:10.1371/journal.pgen.1004044)
Supplement: Table S6 — Reproductive performance of nine subfertile animals not attributable to the c.483C>A - mutation. Inseminations resulting in progeny were considered as successful. (PDF) [file pgen.1004044.s019.pdf]

Animal ID	Birth year	Male reproductive ability	Non-return-rate 56 (cows) %	Number of inseminations	Number of successful inseminations	Proportion of successful inseminations
Subfertile_2 1	2003	-20	37.4	998	69	0.0691
Subfertile_2 3	1999	-26	34.8	1178	67	0.0569
Subfertile_2 2	2000	-24	34.8	1927	105	0.0545
Subfertile_2 4	2004	-24	31.9	1692	74	0.0437
Subfertile_2 5	2003	-23	45.4	1215	244	0.2008
Subfertile_2 6	2008	-23	33.4	862	119	0.1381
Subfertile_2 7	2007	-21	44.8	1183	207	0.1750
Subfertile_2 8	2008	-28	26.7	629	7	0.0111
Subfertile_2 9	2009	-21	34.5	981	119	0.1213
Ø 9 subfertile animals		-23.33	35.9	1185	112.3	0.0967
