# Supplementary material for: A Nonsense Mutation in TMEM95 Encoding a Nondescript Transmembrane Protein Causes Idiopathic Male Subfertility in Cattle
Source: PLoS Genet. 2014 Jan 2;10(1):e1004044. doi: 10.1371/journal.pgen.1004044 (PMC3879157; doi:10.1371/journal.pgen.1004044)
Supplement: Table S7 — Primers and probes used for TaqMan genotyping assays. (PDF) [file pgen.1004044.s020.pdf]

NCBI rsSNP ID	Gene	Primer sequence (5' > 3')	Probe sequence (5' > 3')
rs378652941	*TMEM95*	F:CTTTGGGAAGCTCGGATCCT<br>R:CTCGGGACACCCAGGAG	VIC:AGGGCAGTTCCGCACAC<br>FAM:AGGGCAGTTCCTCACAC
rs381722524	*KIF1C*	F:CGATACACCTGCTGCTGAGA<br>R:GGGTTCCTGCTGTCTCTCTTG	VIC:ACCCCCAGTTTGCA<br>FAM:CCCCCGGTTTGCA
rs385135118	*ACADVL*	F:CAGCGTCCATCCGATCCT<br>R:TCTTGCTTCCGTTGAGGGTATAGTA	VIC:TGTGCCCAGCCCCTGT<br>FAM:TGCCCAGCACCTGT
